# Replacement of dietary saturated with unsaturated fatty acids is associated with beneficial effects on lipidome metabolites: a secondary analysis of a randomized trial

**DOI:** 10.1016/j.ajcnut.2023.03.024

**Published:** 2023-04-11

**Authors:** Laury Sellem, Fabian Eichelmann, Kim G. Jackson, Clemens Wittenbecher, Matthias B. Schulze, Julie A. Lovegrove

**Affiliations:** 1 Hugh Sinclair Unit of Human Nutrition, and Institute for Cardiovascular and Metabolic Research, Department of Food and Nutritional Science, University of Reading, Whiteknights, Pepper Lane, Harry Nursten Building, Reading, UK; 2 Department of Molecular Epidemiology, German Institute of Human Nutrition Potsdam-Rehbruecke, Nuthetal, Germany; 3 German Center for Diabetes Research (DZD), München-Neuherberg, Germany; 4 Division of Food Science and Nutrition, Department of Biology and Biological Engineering, Chalmers University of Technology, Gothenburg, Sweden; 5 Institute of Nutritional Science, University of Potsdam, Nuthetal, Germany

**Keywords:** dietary fat, lipidomics, cardiovascular disease, type 2 diabetes, randomized controlled trial, EPIC-Potsdam

## Abstract

**Background:**

The effects of replacing dietary saturated fatty acids (SFAs) with monounsaturated fatty acids (MUFAs) and/or polyunsaturated fatty acids (PUFAs) on the plasma lipidome in relation to the cardiometabolic disease (CMD) risk is poorly understood.

**Objectives:**

We aimed to assess the impact of substituting dietary SFAs with unsaturated fatty acids (UFAs) on the plasma lipidome and examine the relationship between lipid metabolites modulated by diet and CMD risk.

**Methods:**

Plasma fatty acid (FA) concentrations among 16 lipid classes (within-class FAs) were measured in a subgroup from the Dietary Intervention and VAScular function (DIVAS) parallel randomized controlled trial (*n* = 113/195), which consisted of three 16-wk diets enriched in SFAs (target SFA:MUFA:n-6PUFA ratio = 17:11:4% total energy [TE]), MUFAs (9:19:4% TE), or a MUFA/PUFA mixture (9:13:10% TE). Similar lipidomics analyses were conducted in the European Prospective Investigation into Cancer and Nutrition (EPIC)-Potsdam study (specific case/cohorts: *n* = 775/1886 for type 2 diabetes [T2D], *n* = 551/1671 for cardiovascular disease [CVD]). Multiple linear regression and multivariable Cox models identified within-class FAs sensitive to replacement of dietary SFA with UFA in DIVAS and their association with CMD risk in EPIC-Potsdam. Elastic-net regression models identified within-class FAs associated with changes in CMD risk markers post–DIVAS interventions.

**Results:**

DIVAS high-UFA interventions reduced plasma within-class FAs associated with a higher CVD risk in EPIC-Potsdam, especially SFA-containing glycerolipids and sphingolipids (e.g., diacylglycerol (20:0) *z*-score = −1.08; SE = 0.17; *P* value < 10^−8^), whereas they increased those inversely associated with CVD risk. The results on T2D were less clear. Specific sphingolipids and phospholipids were associated with changes in markers of endothelial function and ambulatory blood pressure, whereas higher low-density lipoprotein cholesterol concentrations were characterized by higher plasma glycerolipids containing lauric and stearic acids.

**Conclusions:**

These results suggest a mediating role of plasma lipid metabolites in the association between dietary fat and CMD risk. Future research combining interventional and observational findings will further our understanding of the role of dietary fat in CMD etiology.

This trial was registered in ClinicalTrials.gov as NCT01478958.

## Abbreviations

Ach: acetylcholine
AU: arbitrary units
CE: cholesteryl ester
CER: ceramides
CLP: Complex Lipid Platform
CMD: cardiometabolic disease
DBP: diastolic blood pressure
DIVAS: Dietary Intervention and VAScular function
EPIC: European Prospective Investigation into Cancer and Nutrition
FMD: flow-mediated dilation
HCER: hexosylceramide
LDI: laser Doppler imaging microvascular response
LPC: lysophosphatidylcholine
LPE: lysophosphatidylethanolamine
MAG: monoacylglycerol
NOx: nitrogen oxides
PC: phosphatidylcholine
PE: phosphatidylethanolamine
PEP: phosphatidylethanolamine plasmalogen
PP: pulse pressure
QUICKI: quantitative insulin sensitivity check index
RCT: randomized controlled trial
rQUICKI: revised quantitative insulin sensitivity index
SBP: systolic blood pressure
SNP: sodium nitroprusside
T2D: type 2 diabetes
TG: triglycerides
UFA: unsaturated fatty acid
VCAM-1: vascular cell adhesion protein 1.

## Introduction

Reducing dietary SFAs has been proposed as a modifiable dietary strategy to help prevent cardiometabolic disease (CMD) risk worldwide [1]. In particular, the replacement of dietary SFAs with unsaturated fatty acids (UFAs) confers a greater benefit than other nutrients (i.e., proteins or carbohydrates) on markers of CVD risk [2,3]. However, it is unclear whether such cardioprotective effects might stem from UFAs themselves, from reduced intakes of deleterious SFAs, or from a combination of both.

Plasma lipids mostly derive from nutrient intakes, along with lipids stored and/or endogenously synthesized in the liver and adipose tissue [4]. In particular, lipids can be solubilized in plasma by binding with proteins, such as free fatty acids with albumins, or as more complex lipids and lipoproteins [5]. More than 600 lipid molecular species have been identified in plasma using high-throughput analytical methods such as lipidome-wide screenings, which differ in their structures and physiological functions [6]. For instance, glycerolipids are characterized by the presence of a glycerol backbone bonding fatty acids (e.g., in mono-, di-, and triacylglycerols), along with a phosphate-containing polar head in glycerophospholipids. Other plasma lipid classes include sphingolipids, such as sphingomyelin and ceramides, and sterols, which are important elements to cellular membrane structure [4]. Complex plasma lipid structures, such as LDL or TG, are important in the mechanistic link between diet and CMD risk [7,8]. Further hypotheses on the involvement of plasma lipid molecular species in cardiometabolic health have been generated, thanks to the growing popularity of high-throughput lipidomics analyses [[9], [10], [11], [12], [13]]. Nonetheless, little is known on the impact of manipulating dietary fat intakes on the plasma lipidome and how this may mediate the epidemiological associations observed between dietary fat intakes and CMD risk.

Methodological constraints have been important obstacles to the assessment of the causal relationship between dietary intakes, CMD risk markers, and CMD events. To date, established knowledge on the role of dietary fat on CMD risk derives from interventional studies such as randomized controlled trials (RCTs), which usually investigate the direct effect of dietary fat on short- to medium-term changes in CMD risk markers, to observational prospective cohort studies, which allow for the long-term follow-up necessary to observe CMD events in large populations. Therefore, there is a need for intersectional research that would provide novel insights into CMD etiology. To that end, recent analysis of the European Prospective Investigation into Cancer and Nutrition (EPIC)-Potsdam study investigated lipidome-wide CMD association analyses across different lipidomics levels and identified direct associations between SFA-containing glycerolipids in plasma and CVD or type 2 diabetes (T2D) incidence, along with direct associations between ceramides and CVD risk [13]. In addition, some of the lipid metabolites associated with T2D and CVD in the EPIC-Potsdam study may have been modulated by dietary fat intakes in a subset of participants from the Dietary Intervention and VAScular function (DIVAS) RCT [14]. Using a different approach and to clarify these potential relationships, this secondary analysis of the DIVAS RCT and the EPIC-Potsdam study identified specific lipidomic species modulated by the dietary exchange of SFAs with UFAs before determining the associations with T2D and CVD outcomes in the EPIC-Potsdam study. To provide a mechanistic insight, changes in DIVAS CMD endpoint risk markers (e.g., fasting lipid profiles and markers of vascular function and inflammation) after 16-wk dietary fat intervention were associated with changes in within-class FAs.

## Methods

### The Dietary Intervention and VAScular function randomized controlled trial

A random selection of participants from each of the three intervention arms (broadly matched for age, sex, and BMI) from the DIVAS RCT with blood samples available at baseline and 16 wk post intervention was included in this secondary analysis (*n* = 113. Table 1). The protocol of the DIVAS study was previously described in detail [14]. The study was conducted according to the guidelines of the Declaration of Helsinki, and ethical approval was obtained from the West Berkshire Local Research Ethics Committee (09/H0505/56) along with the University of Reading Research Ethics Committee (09/40). All participants provided their written informed consent before inclusion. Briefly, the DIVAS study was a 16-wk, single-blind, parallel RCT conducted between 2009 and 2012 (www.clinicaltrials.gov; NCT01478958), which enrolled nonsmoking males and females from the Berkshire (UK) area, aged 21–60 y, with a moderate CVD risk identified via a scoring tool developed by Weech et al. [15]. Inclusion criteria also included normal fasting lipid profiles, kidney and liver functions, no history of CVD or diabetes in the year preceding inclusion, no medication for hypertension, no pregnancy or lactation, and no excessive physical activity levels (>3 sessions of 20 min/wk) or alcohol consumption (≤14 units/wk for females and ≤21 units for males, corresponding to the recommended alcohol intakes at the time the study was conducted).

**TABLE 1:** Preintervention characteristics and cardiometabolic disease risk markers in the subset of participants from the DIVAS randomized controlled trial (*n* = 113).

		Dietary intervention group		
	Overall *n* = 113	SFA-rich *n* = 38	MUFA-rich *n* = 39	MUFA/PUFA-rich *n* = 36
	Mean (SD)	Mean (SD)	Mean (SD)	Mean (SD)
Age, y	43.8 (10.2)	46.1 (7.4)	42.2 (12.1)	43.3 (10.2)
Sex, *n* (%)				
Female	68 (60.2%)	21 (55.3%)	23 (59.0%)	24 (66.7%)
Male	45 (39.8%)	17 (44.7%)	16 (41.0%)	12 (33.3%)
BMI, kg/m^2^	26.8 (4.3)	27.7 (4.9)	26.1 (4.0)	26.5 (3.7)
Fasting lipid markers				
Total cholesterol, mmol/L	5.52 (1.12)	5.54 (1.09)	5.35 (0.99)	5.68 (1.28)
LDL cholesterol, mmol/L	3.79 (1.02)	3.80 (1.04)	3.65 (0.85)	3.93 (1.16)
HDL cholesterol, mmol/L	1.49 (0.37)	1.47 (0.34)	1.47 (0.39)	1.52 (0.38)
Total cholesterol:HDL cholesterol ratio	3.91 (1.15)	3.99 (1.30)	3.82 (0.99)	3.94 (1.15)
TG, mmol/L	1.24 (0.70)	1.35 (0.89)	1.18 (0.58)	1.19 (0.58)
NEFAs, μmol/L	471 (171)	484 (121)	446 (179)	484 (207)
Markers of glycemic control				
Glucose, mmol/L	5.00 (0.44)	5.04 (0.45)	4.95 (0.44)	5.01 (0.44)
Insulin, pmol/L	28.0 (15.7)	31.7 (20.2)	27.1 (11.2)	25.2 (13.8)
HOMA-IR	1.05 (0.61)	1.22 (0.81)	0.99 (0.41)	0.94 (0.52)
QUICKI	0.40 (0.04)	0.39 (0.06)	0.39 (0.03)	0.40 (0.04)
rQUICKI	0.46 (0.06)	0.46 (0.08)	0.46 (0.05)	0.47 (0.06)
Markers of endothelial function and arterial stiffness				
FMD, %	5.66 (2.87)	5.44 (2.74)	5.27 (2.57)	6.38 (3.29)
Preocclusion artery diameter, mm	3.86 (0.64)	3.99 (0.67)	3.80 (0.65)	3.78 (0.59)
LDI Ach, AU	1583 (872)	1643(1072)	1555 (734)	1534 (742)
LDI SNP, AU	1491 (683)	1552 (748)	1513 (708)	1383 (563)
Reflection index, %	62.7 (12.7)	65.2 (12.4)	61.1 (12.5)	61.6 (13.0)
Pulse wave velocity, m/s	6.89 (1.19)	7.12 (1.02)	6.53 (1.20)	7.05 (1.25)
Augmentation index, %	23.6 (12.2)	24.8 (10.9)	21.9 (13.5)	24.1 (12.2)
Stiffness index, m/s	6.72 (1.81)	6.98 (2.01)	6.35 (1.34)	6.83 (1.99)
Markers of ambulatory blood pressure				
24-h SBP, mm Hg	121 (11)	120 (12)	119 (9)	124 (11)
24-h DBP, mm Hg	74 (7)	75 (9)	73 (7)	76 (7)
24-h PP, mm Hg	47 (7)	46 (7)	46 (6)	49 (6)
24-h heart rate, beats/min	71 (7)	69 (7)	72 (7)	72 (6)
Day SBP, mm Hg	125 (12)	124 (12)	123 (10)	129 (12)
Day DBP, mm Hg	78 (8)	77 (8)	76 (8)	79 (9)
Day PP, mm Hg	48 (7)	47 (7)	47 (7)	50 (7)
Day heart rate, beats/min	73 (8)	71 (7)	75 (8)	74 (7)
Night SBP, mm Hg	106 (12)	106 (14)	105 (10)	109 (11)
Night DBP, mm Hg	63 (7)	64 (8)	61 (6)	65 (7)
Night PP, mm Hg	47 (7)	46 (7)	46 (6)	49 (6)
Night heart rate, beats/min	62 (6)	62 (7)	62 (6)	63 (6)
Markers of inflammation and endothelial activation				
CRP, mg/L	2.15 (3.29)	2.95 (4.40)	1.59 (2.61)	1.90 (2.41)
NOx, μmol/L	27.5 (17.8)	32.5 (24.9)	23.6 (9.5)	26.4 (14.7)
VCAM-1, ng/mL	670 (171)	662 (142)	670 (199)	676 (172)
ICAM-1, ng/mL	218 (53)	221 (58)	216 (42)	217 (60)
IL-6, pg/mL	1.44 (1.06)	1.85 (1.29)	1.12 (0.78)	1.38 (0.94)
TNF-α, pg/mL	1.17 (0.74)	1.42 (1.01)	1.07 (0.62)	1.00 (0.37)
E-selectin, ng/mL	34.6 (14.7)	34.9 (14.5)	33.9 (12.7)	35.1 (17.0)
P-selectin, ng/mL	41.7 (12.9)	43.7 (12.9)	43.1 (13.3)	38.0 (12.0)
Von Willebrand factor, μU/mL	817 (409)	905 (492)	813 (368)	728 (339)
Urinary microalbumin, mg/24 h	3.56 (6.47)	2.94 (2.48)	1.99 (1.22)	5.72 (10.63)

Abbreviations: AU, arbitrary unit; DBP, diastolic blood pressure; FMD, flow-mediated dilation; ICAM-1, intercellular adhesion molecule-1; LDI Ach, laser Doppler imaging microvascular response to acetylcholine; LDI SNP, laser Doppler imaging microvascular response to sodium nitroprusside; NOx, nitrogen oxides; PP, pulse pressure; QUICKI, quantitative insulin sensitivity check index; rQUICKI, revised quantitative insulin sensitivity index; SBP, systolic blood pressure; TG, triglycerides; VCAM-1, vascular cell adhesion protein 1.

Upon inclusion, participants were randomly allocated to one of the three 16-wk isoenergetic, high-fat (36% TE) dietary intervention arms varying in proportions of SFAs, MUFAs, and PUFAs: a SFA-rich diet (target SFA:MUFA:n-6 PUFA ratio = 17:11:4 % TE), a MUFA-rich diet (target SFA:MUFA:n-6 PUFA ratio = 9:19:4 % TE), or a MUFA/PUFA-rich diet (target SFA:MUFA:n-6 PUFA ratio = 9:13:10 % TE) [15]. The analysis of 4-d weighed diet diaries at baseline and postintervention confirmed participants’ compliance and the successful isoenergetic exchange of dietary fat in each dietary intervention arm [15]*.* In addition, measurements for a wide range of CMD risk markers were performed at baseline (Table 1) and postintervention.

### EPIC-Potsdam study

The EPIC-Potsdam is a prospective cohort study that enrolled 27,548 participants from the Potsdam (Germany) area between 1994 and 1998. The study and recruitment protocols have been previously published elsewhere [13,16,17]. The study received ethical approval from the Medical Society of the State of Brandenburg Ethics Committee, and all participants provided their written informed consent before inclusion. Cases of incident CMD were self-reported by participants during the active follow-up or obtained from death certificates. Additional information on T2D incidence was obtained using self-reported T2D medication or T2D-related dietary modifications, along with evidence of T2D detected in clinical files and death certificates originally related to other pathologies. Participants’ treating physicians were then requested to validate each case and diagnosis date, following guidelines from ICD-10 [18]. CMD considered for analyses included T2D (ICD-10 code: E11), along with fatal or nonfatal cases of primary CVD, which included myocardial infarction (ICD-10 code: I21) or stroke (ICD-10 codes: I63.0 to I63.9 for ischemic stroke, I61.0 to I61.9 for intracerebral hemorrhage, I60.0 to I60.9 for subarachnoid hemorrhage, and I64.0 to I64.9 for unspecified stroke).

To assess the associations between molecular phenotypes and T2D or CVD risks, 2 nested case-cohorts were constructed among the participants who provided blood samples at baseline (*n* = 26,437). The construction protocol of the nested case-cohorts has been previously described in detail [13]. In summary, a random subcohort (*n* = 1262) was selected as a reference for both T2D and CVD risks, while incident cases of T2D (censoring date: 30 November, 2006) or CVD (censoring date 31^st^ August 2005) were drawn from the overall EPIC-Potsdam cohort. Participants without follow-up or suffering from prevalent T2D or CVD were excluded from the analysis. The final T2D nested case-cohort included *n* = 1886 participants (among which *n* = 775 T2D cases), while the CVD nested case-cohort included *n* = 1671 participants (among which *n* = 551 CVD cases).

### Lipidomics analyses

Fasted blood plasma samples were collected in citrated vacutainer collection tubes at baseline (week 0) and postintervention (week 16) from participants from the DIVAS study and stored at −80 °C until analysis. Citrated plasma samples from the DIVAS study participants (*n* = 113) were analyzed by Metabolon Inc., who performed a Complex Lipids Platform™ (CLP) analysis to measure absolute concentrations of 987 molecular species (in μmol/L). Similar analyses were performed on citrated plasma samples from the baseline assessment in the EPIC-Potsdam study [13]. Briefly, plasma lipids were extracted using a butanol:methanol (BUME) solution as described by Löfgren et al. [19], concentrated under nitrogen and reconstituted in 0.25 mL of 10 mM ammonium acetate dichloromethane:methanol (50:50). Infusion-mass spectrometry analyses were then performed on a Sciex SelexIon 5500 QTRAP mass spectrometer. To determine characteristics fragments and lipid concentrations, analyses were performed in multiple reaction monitoring mode and samples were injected with deuterated internal standards. Metabolon Inc. reported mean relative SD of <5% for all measured lipid species.

### Statistical analysis

All statistical analyses were performed in R (version 4.1.2). All statistical tests were 2-sided, and *P* values of <0.05 were considered statistically significant.

#### Computation of within-class FA concentrations

Missing values in molecular species concentrations were imputed using the “Quantile Regression Imputation of Left-Censored data” method from the imputeLCMD R package [20], except for molecular species with ≥75% of missing values, which were excluded from analyses.

The CLP analysis measured absolute concentrations of lipid molecular species across lipid classes classified as neutral lipids, sphingolipids, and phospholipids, all of which contain between 1 and 3 FAs within their molecular structure. Neutral lipids included cholesteryl esters (CEs), monoacylglycerols (MAGs), diacylglycerols (DAGs), and TGs. Sphingolipids included sphingomyelins (SMs), ceramides (CERs), hexosylceramides (HCERs), lactosylceramides (LCERs), and dihydroceramides (DCERs). Finally, phospholipids included phosphatidylcholine (PC), lysophosphatidylcholine (LPC), phosphatidylinositol (PI), phosphatidylethanolamine (PE), phosphatidylethanolamine ether (PEO), phosphatidylethanolamine plasmalogen (PEP), and lysophosphatidylethanolamine (LPE). Within each lipid class, molecular species containing a specific FA were summed to compute within-class FA concentrations. Thus, for lipid classes containing one FA per molecule (i.e., CE, MAG, CER, DCER, LCER, HCER, SM, SPE, and LPC), within-class FA and molecular species concentrations were equivalent. Lipid classes containing 2 or 3 FAs per molecule (i.e., DAG, TG, PC, PE, PEO, PEP, and PI) were accounted for by weighing their contribution to a specific within-class FA using the amount of FA of interest present in the molecule (e.g., a DAG molecule containing 2 palmitic acids [16:0] contributed twice to the calculation of the DAG [16:0] within-class FA concentration, while a DAG molecule containing a palmitic acid [16:0] and a myristic acid [14:0] contributed once to the DAG [16:0] and once to the DAG [14:0] within-class FA concentrations). Finally, the total lipid class concentrations were calculated by summing all molecular species concentrations from the same lipid class. For conciseness and legibility, within-class FAs will be referred to as their shorthand notations thereafter (e.g., DAG [16:0]). A list of the full names of all FAs investigated in this study is available in Supplemental Table 1.

#### Effects of the DIVAS dietary fat intervention on within-class FA plasma concentrations

The effects of the 2 UFA-rich diets (MUFA-rich and MUFA/PUFA-rich) from the DIVAS RCT on post intervention within-class FA concentrations were assessed using multiple linear regression models adjusted for age, BMI, sex, and baseline concentration of the within-class FA of interest, along with baseline and postintervention concentration of the total lipid class of interest. All within-class FA concentrations were log-transformed and *z* normalized (mean = 0; SD = 1) to allow comparison of the effect size across within-class FA concentrations and account for potentially skewed distributions. Dietary intervention arms were coded as dummy variables to express linear regression model results as changes in *z*-score in the MUFA-rich and MUFA/PUFA-rich diets compared to the SFA-rich diet (used as reference). To account for multiple testing, *P* values from multiple linear regression models were adjusted using the Bonferroni correction method [21].

All models were checked for linearity using scatter plots, for normality of residuals using QQ-plots, and for homogeneity of variance and independence of residuals using residuals plotted against predicted or observed outcome values, respectively.

#### Associations between within-class FA plasma concentrations and changes in CMD risk markers in DIVAS

Elastic-net linear regression (ENR) models were conducted to identify and select changes in within-class FA concentrations associated with changes in individual CMD risk markers (dependent variable) measured during the DIVAS RCT. ENR approaches have been successfully implemented in recent nutritional epidemiological research for the identification of simple and predictive models among omics datasets, which often present high dimensionality and risk of collinearity [[22], [23], [24]].

In this analysis, we adapted the method described by Drouin-Chartier et al. [22] to perform ENR analyses using the *glmnet* R package [25]. Briefly, all within-class FA concentrations and CMD risk markers were expressed as changes from baseline (week 16 − week 0), and within-class FA concentrations were *z* normalized (mean = 0; SD = 1) to ensure comparability of effect sizes. However, due to the presence of negative changes from baseline, variables could not be log-transformed. First, a 10-fold crossvalidation (CV) approach (training/testing sets: 80%/20% of the data, respectively) was used to determine the penalty (α) and tuning parameters (λ), which would optimize the complexity of the model (i.e., number of within-class FAs selected) while minimizing the mean-squared error between the measured and predicted changes in the dependent variable and mitigating risk of overfitting. The chosen α and λ parameters were then used to compute ENR models in a separate 10-fold CV procedure similar to the one described above, from which we extracted the list of every within-class FAs selected in each of the 10 iterations along with their estimated regression coefficients. To build the final model, we considered within-class FAs consistently selected in the 10-fold CV procedure (i.e., in at least 9 iterations out of 10), and averaged their regression coefficients from each iteration to obtain a final, unique coefficient for each retained within-class FA. Finally, the performance of this approach was evaluated by comparing the predicted dependent variable values (i.e., CMD risk markers) to the actual ones measured during the DIVAS study. To do this, within each 10-fold CV iteration, the model obtained from the training set was fitted to its respective testing set to compute the predicted values of the dependent variable. The subsets from each iteration were then collated into one dataset, and the overall Pearson correlation between the predicted and measured values was used as a proxy for performance.

This method was repeated for each dependent variable of interest, which consisted of 41 CMD risk markers measured in the DIVAS study (Table 1), to obtain predictive models based on within-class FA plasma concentrations.

Finally, the associations between within-class FA concentrations and CMD risk markers identified by ENR analyses were further tested in fully adjusted multiple linear regression models, including age (continuous; in years), sex (female/male), BMI (continuous; kg/m^2^), baseline CMD risk marker value (continuous; units in Table 1), and DIVAS dietary intervention group (SFA-rich, MUFA-rich, or MUFA/PUFA-rich diet).

#### Associations between within-class FA plasma concentrations and CMD risk in EPIC-Potsdam

To contextualize the observed dietary effects, we determined whether all lipids that significantly changed in response to replacing SFA with UFA in the DIVAS study were associated with CMD risk in the EPIC-Potsdam study. HRs for the associations between within-class FA concentrations and incident risks of CVD or T2D were computed using multivariable proportional hazards Cox models, adjusted for age (timescale), sex, waist circumference (data not shown), height, leisure time physical activity, smoking status, alcohol intake, highest achieved education level, fasting status as blood draw, total energy intake, diastolic and SBPs, circulating total cholesterol, HDL cholesterol, and TG concentrations, antihypertensive medication, lipid-lowering medication, and acetylsalicylic acid medication. In addition, each model was adjusted for the concentration of the total lipid class to which the within-class FA concentration of interest belonged (e.g., models on DAG [16:0] were adjusted for total DAG plasma concentration). All within-class FA concentrations were log-transformed and *z* normalized (mean = 0; SD = 1) to allow comparison of the effect size across within-class FA concentrations and account for potentially skewed distributions. Participants contributed to each model until the date of diagnosis, death, loss to follow-up, or censoring date, whichever occurred first. In addition, the case-cohort design and multiple testing were accounted for using assigned weights as proposed by Prentice [26] and the false discovery rate method [27], respectively. Inspection of Schoenfeld residuals per lipid metabolite indicated no violation of the proportional hazards assumption.

## Results

The flow chart of participants included in the DIVAS study and the current secondary analysis is presented in Supplemental Figure 1. Baseline characteristics of the DIVAS study participant subset (*n* = 113) according to their allocated dietary intervention group are presented in Table 1. Within the included participants, 60.2% were females, the overall mean age was 44 y (SD = 10.2), and the overall mean BMI was 26.8 kg/m^2^ (SD = 4.3). Baseline characteristics of the participants from the EPIC-Potsdam study have been previously published [13].

**FIGURE 1:**
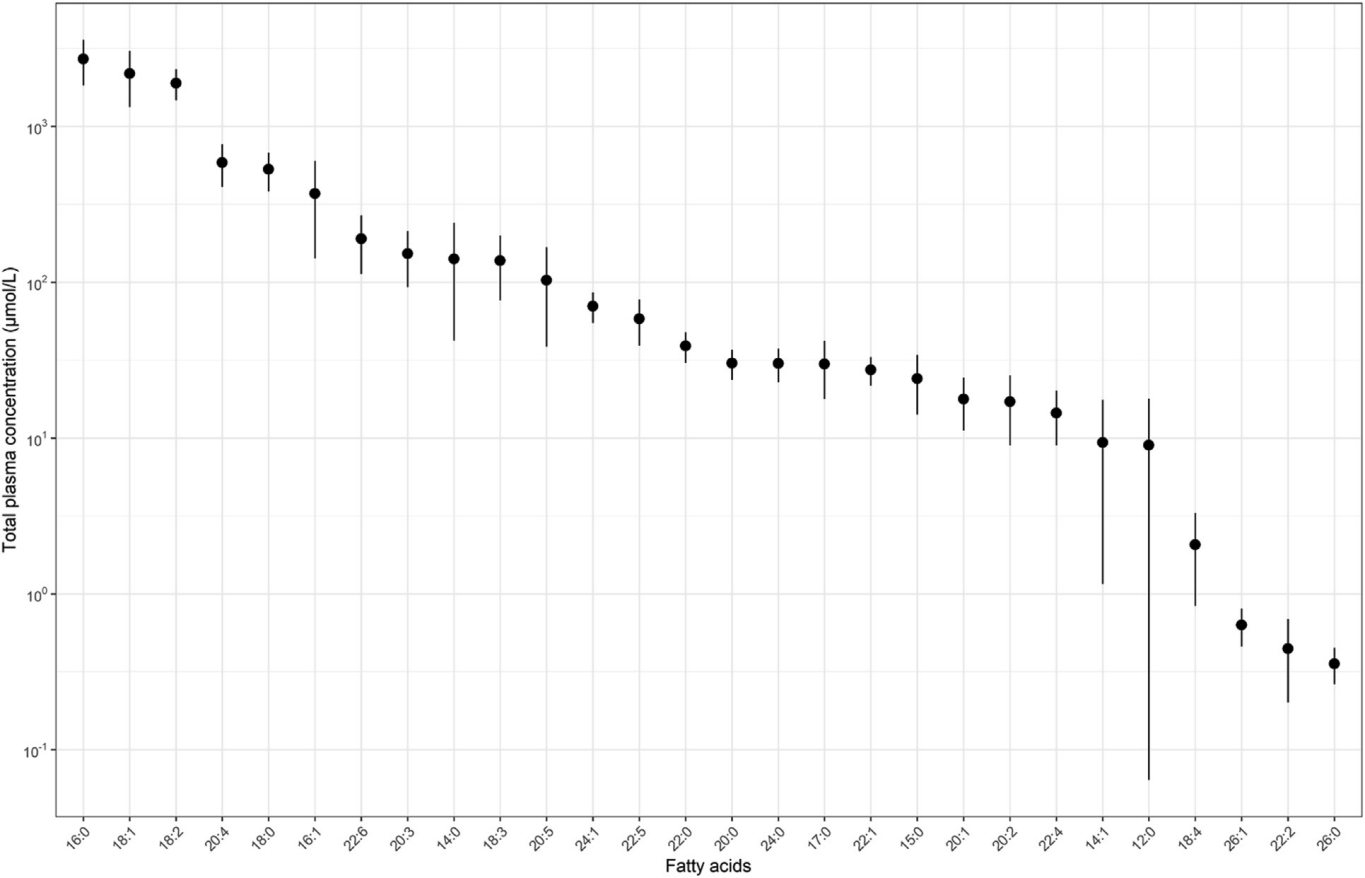
Total plasma concentrations of 28 fatty acids identified in lipidome-wide screening among participants from the DIVAS study prior to the start of the dietary intervention (*n* = 113). Full names of fatty acids are listed in Supplemental Table 1. Data points are represented as mean and SD.

**FIGURE 2:**
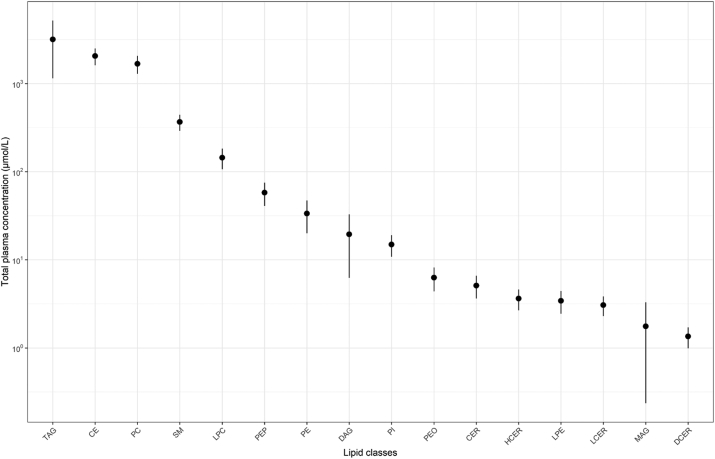
Total plasma concentrations of 14 lipid classes identified in lipidome-wide screening among participants from the DIVAS study prior to the start of the dietary intervention (*n* = 113). Data points represented as mean and SD. CE, cholesteryl esters; CER, ceramides; DAG, diacylglycerol; DCER, dihydroceramide; HCER, hexosylceramide; LCER, lactosylceramide; LPC, lysophosphatidylcholine; LPE, lysophosphatidylethanolamine; MAG, monoacylglycerol; PC, phosphatidylcholine; PE, phosphatidylethanolamine; PEO, phosphatidylethanolamine ether; PEP, phosphatidylethanolamine plasmalogen; PI, phosphatidylinositol; SM, sphingomyelins.

**FIGURE 3:**
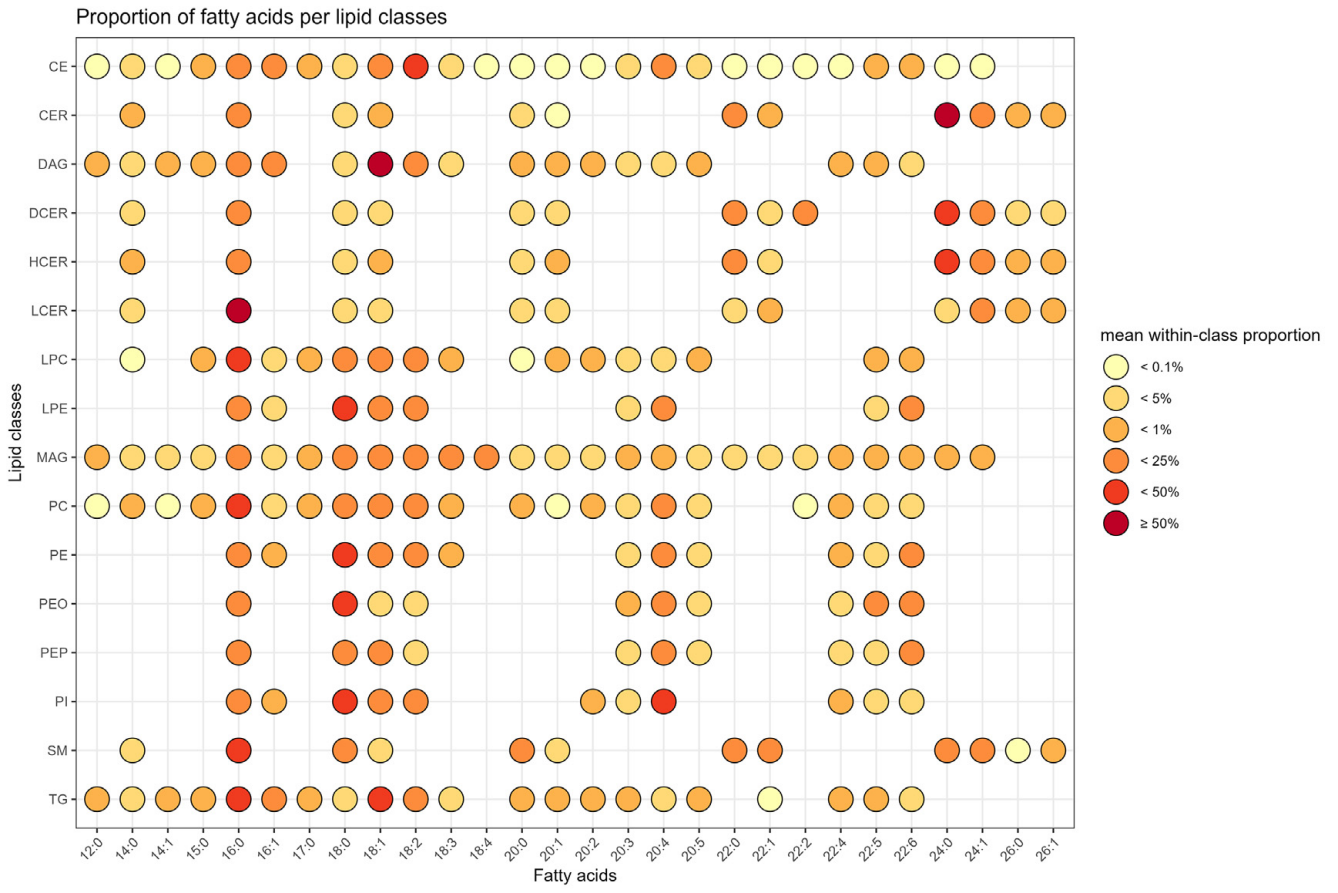
Proportion of fatty acids in plasma lipid classes among participants from the DIVAS study prior to the start of the dietary intervention (*n* = 113). Full names of fatty acids are listed in Supplemental Table 1. CE, cholesteryl ester; CER, ceramide; DAG, diacylglycerol; DCER, dihydroceramide; HCER, hexosylceramide; LCER, lactosylceramide; LPC, lysophosphatidylcholine; LPE, lysophosphatidylethanolamine; MAG, monoacylglycerol; PC, phosphatidylcholine; PE, phosphatidylethanolamine; PEO, phosphatidylethanolamine ether; PEP, phosphatidylethanolamine plasmalogen; PI, phosphatidylinositol; SM, sphingomyelin.

The CLP analysis identified a total of 987 molecular species in plasma samples, of which 101 were excluded from further analyses due to the high proportion of missing values (≥75%). Among the 886 molecular species retained, 28 different FAs were detected (Supplemental Table 1), and a total of 243 within-class plasma FA concentrations across 16 total lipid classes were available for analyses. In plasma samples from the DIVAS participants prior to dietary intervention, palmitic (16:0) and oleic (18:1) acids were the most abundant FAs across all lipid classes (Figure 1), whereas TG and CE were the most abundant lipid classes (Figure 2). As presented in Figure 3, 16:0 was the most abundant FA in sphingolipids (LCER, SM) and some phospholipids (LPC and PC), whereas stearic acid (18:0) accounted for the majority of FAs in most phospholipids (LPE, PE, PEO, PEP, and PI). Other ceramides (CER, DCER, and HCER) mostly contained lignoceric acid (24:0). Finally, 18-carbon UFAs were the most abundant FAs in CE (i.e., 18:2) and MAG (i.e., 18:4), followed by DAG and TG (i.e., 18:1).

### Effects of the DIVAS dietary fat intervention on within-class FA plasma concentrations

**FIGURE 4:**
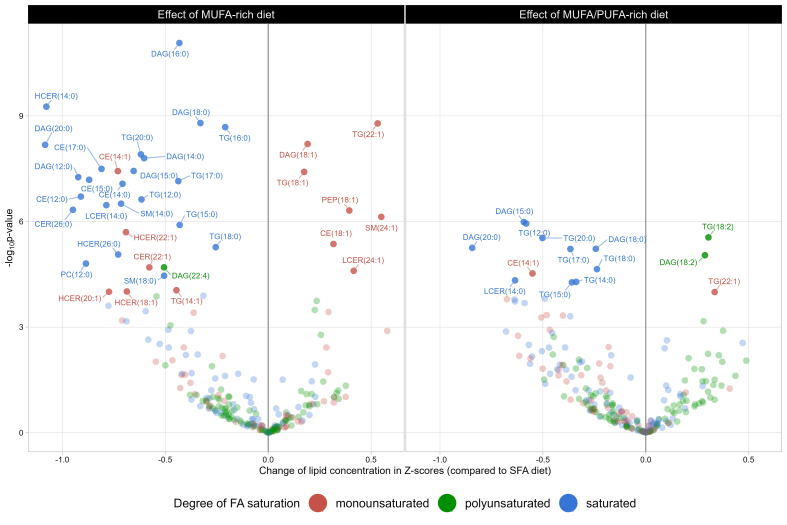
Effect of MUFA-rich and MUFA/PUFA–rich dietary interventions compared with a SFA-rich diet on plasma lipid metabolites identified in lipidome-wide screening among participants from the DIVAS study (*n* = 113). Assessed using multiple linear regression models adjusted for age, BMI, sex, and baseline concentration of the within-class fatty acid of interest, along with baseline and postintervention concentration of the total lipid class of interest. *P* values were adjusted for multiple testing using the Bonferroni correction method (0.05/282 = 0.00018). Unlabeled data points represent within-class fatty acid concentrations not significantly affected by the DIVAS dietary intervention after Bonferroni correction (*P* value ≥ 0.05). CE, cholesteryl ester; CER, ceramide; DAG, diacylglycerol; DCER, dihydroceramide; HCER, hexosylceramide; LCER, lactosylceramide; LPC, lysophosphatidylcholine; LPE, lysophosphatidylethanolamine; MAG, monoacylglycerols; PC, phosphatidylcholine; PE, phosphatidylethanolamine; PEO, phosphatidylethanolamine ether; PEP, phosphatidylethanolamine plasmalogen; PI, phosphatidylinositol; SM, sphingomyelin.

The impact of the 16-wk MUFA and MUFA/PUFA-rich diets compared with that of the SFA-rich diet (as expressed in *z*-scores) on plasma lipid metabolites in participants from the DIVAS study is presented in Figure 4. Overall, 40 lipid metabolites were significantly changed by at least one of the UFA diets in comparison to the SFA-rich diet, 22 were affected by both, 26 exclusively by the MUFA-rich diet, and 3 exclusively by the MUFA/PUFA-rich diet. Relative to the SFA-rich diet, the MUFA-rich diet resulted in lower concentrations of SFAs across most lipid classes, with the largest reductions observed for DAG (20:0; *z*-score = −1.08; SE = 0.17; *P* value < 10^−8^) and HCER (14:0; *z*-score = −1.08; SE = 0.16; *P* value < 10^−9^) plasma levels. Furthermore, the MUFA-rich diet led to higher concentrations of long-chain MUFAs (18:1, 22:1, and 24:1) in CE, DAG, TG, PEP, LCER, and SM than the SFA-rich diet, with the largest increases observed for SM (24:1; *z*-score = 0.55; SE = 0.10; *P* value < 10^−6^) and TG (22:1; *z*-score = 0.53; SE = 0.08; *P* value < 10^−8^) plasma levels. However, some MUFA concentrations were lower following the MUFA-rich diet, such as 22:1 in CER; 14:1 in CE and TG; and 18:1, 20:1 and 22:1 in HCER. Similar albeit less diverse results were observed when comparing the effects of the MUFA/PUFA-rich diet with those of the SFA-rich diet. Specifically, the MUFA/PUFA-rich diet led to higher concentrations of 18:2 in DAG (*z*-score = 0.29; SE = 0.06; *P* value < 10^−5^; data not tabulated) and TG (*z*-score = 0.30; SE = 0.06; *P* value < 10^−5^; data not tabulated), but fewer reductions in SFAs than in the MUFA-rich diet (Figure 4).

### Associations between within-class FA plasma levels and changes in CMD risk markers in DIVAS

**FIGURE 5:**
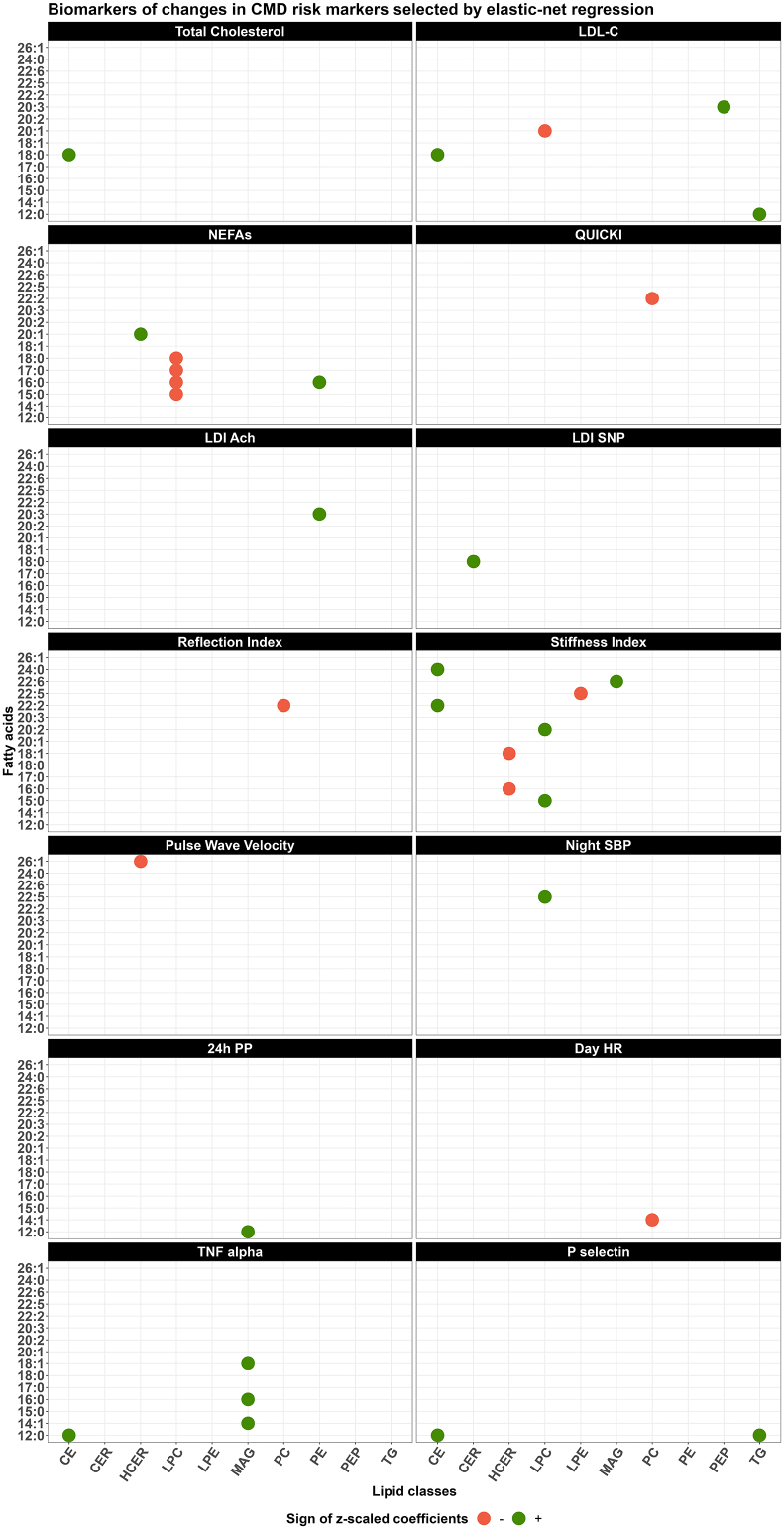
Lipid metabolites associated with changes in cardiometabolic risk markers measured among participants from the DIVAS study (*n* = 113). Identified using 10-fold crossvalidated elastic-net regression models. CE, cholesteryl esters; CER, ceramides; DAG, diacylglycerols; HCER, hexosylceramides; LDI Ach, laser Doppler imaging microvascular response to acetylcholine; LDI SNP, laser Doppler imaging microvascular response to sodium nitroprusside; LDL-C, LDL cholesterol; LPC, lysophosphatidylcholine; LPE, lysophosphatidylethanolamine; MAG, monoacylglycerol; PC, phosphatidylcholine; PE, phosphatidylethanolamine; PEP, phosphatidylethanolamine plasmalogen; PP, pulse pressure; QUICKI, quantitative insulin sensitivity check index; SBP, systolic blood pressure.

A total of 14 out of the 41 CMD risk markers measured in the DIVAS study were linked with changes in within-class FAs using ENR models (Figure 5, Supplemental Figure 2). In particular, increased LDL cholesterol concentrations between post- and preintervention measures were associated with increased plasma TG (12:0), CE (18:0), and PEP (20:3) levels (predictive Pearson correlation = 0.21; Supplemental Table 2), whereas increases in nonesterified FA (NEFA) concentrations were associated with reduced levels of SFAs in plasma LPC (i.e., 15:0, 16:0, 17:0, and 18:0) but higher levels of PE (16:0; predictive Pearson correlation = 0.26, Supplemental Table 2). Furthermore, various changes in within-class FA plasma concentrations were associated with beneficial effects on endothelial function and arterial stiffness estimations, such as the laser Doppler imaging microvascular response to acetylcholine or sodium nitroprusside (LDI Ach and LDI SNP, respectively), reflection and arterial stiffness indexes, and pulse wave velocity. Specifically, a higher arterial stiffness index was related to higher levels of long-chain FAs (i.e., 20:2, 22:2, 24:0, and 22:6) in CE, LPC, or MAG and was associated with lower levels of 16:0 and 18:1 in HCER and LPE (22:5; predictive Pearson correlation = 0.13; Supplemental Table 2). Finally, higher TNF-α concentrations were associated with increased levels of 14:1, 16:0, and 18:1 in MAG and of 12:0 in CE (predictive Pearson correlation = 0.18; Supplemental Table 2). Similarly, higher P-selectin concentrations were related to increased proportions of 12:0 in CE and TG (predictive Pearson correlation = 0.24; Supplemental Table 2).

Results for the multiple linear regression models are presented in Table 2. In fully adjusted models, there were no statistically significant associations between within-class FA concentrations and NEFAs, quantitative insulin sensitivity check index (QUICKI), pulse wave velocity, or TNF-α concentrations. For other CMD risk markers, multiple linear regression models confirmed associations of single within-class FA concentrations with total cholesterol, LDI Ach, LDI SNP, reflection index, night systolic blood pressure (SBP), 24-h pulse pressure (PP), day heart rate levels, and P-selectin. Furthermore, changes in LDL cholesterol concentrations remained positively associated with changes in TG (12:0; (β = 0.15; 95% CI: 0.04, 0.26; *P* value = 0.01) and CE (18:0; β = 0.20, 95% CI: 0.08, 0.32; *P* value < 0.01) concentrations (Table 2). Finally, changes in the arterial stiffness index remained positively associated with changes in plasma concentration of CE (22:2; β = 0.49; 95% CI: 0.10, 0.87; *P* value = 0.01), LPC (15:0; β = 0.34; 95% CI: 0.01, 0.66; *P* value = 0.04), and LPC (20:2; β = 0.45; 95% CI: 0.12, 0.78; *P* value = 0.008; Table 2).

**TABLE 2:** Results from multiple linear regression models on the associations between changes in within-class FA concentrations and CMD risk biomarkers in the DIVAS randomized controlled trial (*n* = 113).^1^

Within-class FA	β estimate 2	95% CI lower-bound	95% CI upper-bound	*P* value	*R*^2^	*n*
Total cholesterol, mmol/L						
CE (18:0)	0.26	0.13	0.39	<0.001	0.24	113
LDL cholesterol, mmol/L						
LPC (20:1)	−0.16	−0.26	−0.06	<0.01	0.41	113
PEP (20:3)	0.03	−0.09	0.14	0.64		
TG (12:0)	0.15	0.04	0.26	0.01		
CE (18:0)	0.20	0.08	0.32	<0.01		
NEFAs, mmol/L						
PE (16:0)	19.83	−8.09	47.75	0.16	0.37	113
HCER (20:1)	26.03	−3.24	55.30	0.08		
LPC (15:0)	−5.46	−45.83	34.91	0.79		
LPC (16:0)	−17.24	−74.48	40.01	0.55		
LPC (17:0)	−45.13	−95.46	5.20	0.08		
LPC (18:0)	3.18	−53.19	59.55	0.91		
QUICKI						
PC (22:2)	−0.003	−0.011	0.004	0.34	0.33	97
LDI Ach, AU						
PE (20:3)	230.83	106.96	354.71	<0.001	0.59	89
LDI SNP, AU						
CER (18:0)	172.20	40.13	304.26	0.01	0.56	89
Reflection index, %						
PC (22:2)	−3.11	−5.31	−0.91	0.006	0.24	108
Arterial stiffness index, m/s						
CE (22:2)	0.49	0.10	0.87	0.01	0.55	109
CE (24:0)	−0.05	−0.47	0.36	0.81		
LPE (22:5)	−0.44	−0.77	−0.10	0.01		
MAG (22:6)	0.12	−0.20	0.43	0.47		
HCER (16:0)	−0.27	−0.66	0.12	0.17		
HCER (18:1)	−0.23	−0.64	0.17	0.26		
LPC (15:0)	0.34	0.01	0.66	0.04		
LPC (20:2)	0.45	0.12	0.78	0.008		
Pulse wave velocity, m/s						
HCER (26:1)	−0.18	−0.37	0.01	0.06	0.32	86
Night SBP, mm Hg						
LPC (22:5)	2.30	0.30	4.31	0.03	0.33	90
24-h PP, mm Hg						
MAG (12:0)	1.92	0.75	3.09	0.002	0.40	81
Day heart rate, beats/min						
PC (14:1)	−2.05	−3.67	−0.43	0.01	0.27	84
TNF-α, pg/mL						
CE (12:0)	0.04	0.00	0.08	0.08	0.36	110
MAG (14:1)	0.04	−0.01	0.09	0.10		
MAG (16:0)	0.04	−0.01	0.08	0.10		
MAG (18:1)	0.03	−0.02	0.08	0.21		
P-selectin, ng/mL						
CE (12:0)	2.41	0.26	4.55	0.03	0.20	112
TG (12:0)	1.56	−0.49	3.62	0.13		

Abbreviations: CE, cholesteryl ester; CER, ceramide; DAG, diacylglycerol; HCER, hexosylceramide; LDI Ach, laser Doppler imaging microvascular response to acetylcholine; LDI SNP, laser Doppler imaging microvascular response to sodium nitroprusside; LPC, lysophosphatidylcholine; LPE, lysophosphatidylethanolamine; MAG, monoacylglycerol; PC, phosphatidylcholine; PE, phosphatidylethanolamine; PEP, phosphatidylethanolamine plasmalogen; PP, pulse pressure; QUICKI, quantitative insulin sensitivity check index; SBP, systolic blood pressure; TG, triglycerides.

1 Multiple linear regression adjusted for age (continuous, in years), sex (female/male), BMI (continuous, kg/m^2^), baseline CMD risk marker value (continuous), and dietary intervention group (SFA-rich, MUFA-rich, or MUFA/PUFA-rich diet).

2 Expressed as a change in CMD risk marker per additional SD of plasma within-class FA concentration (μmol/L).

### Associations between within-class FA plasma concentrations and CMD risk in EPIC-Potsdam

**FIGURE 6:**
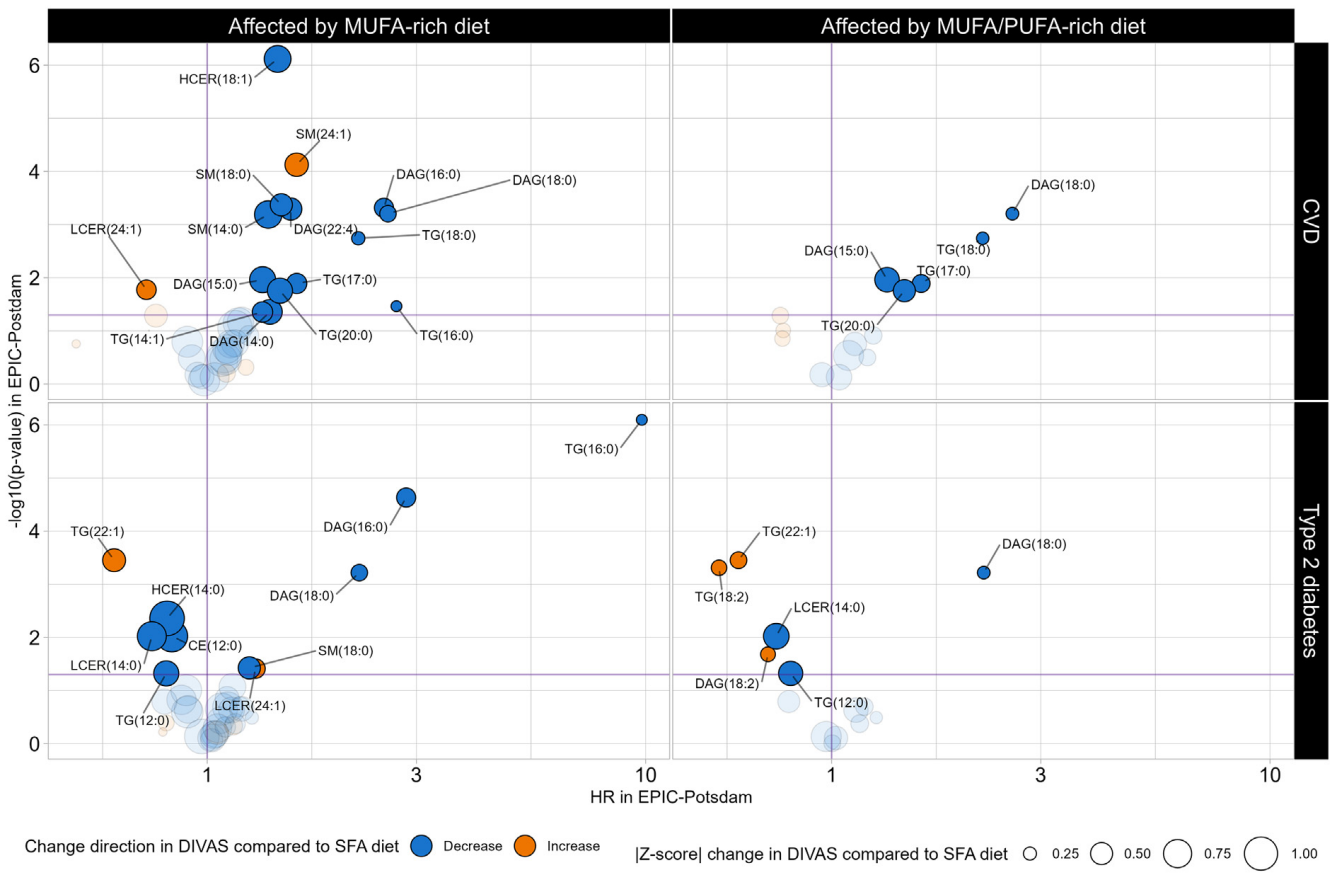
Effect of the DIVAS dietary intervention on lipid metabolites identified in lipidome-wide screening and associations with cardiometabolic disease risk in the EPIC-Potsdam study. Assessed using multivariable Cox proportional hazard models adjusted for age (timescale); sex; waist circumference; height; leisure time physical activity; smoking status; alcohol intake; highest achieved education level; fasting status as blood draw, total energy intake, diastolic and systolic blood pressures, circulating total cholesterol, HDL cholesterol, and TG concentrations; antihypertensive medication; lipid-lowering medication; and acetylsalicylic acid medication. In addition, each model was adjusted for the concentration of the total lipid class to which the within-class FA concentration of interest belonged. *P* values were adjusted for multiple testing using the Bonferroni correction method (0.05/41 = 0.0012). Unlabeled data points represent that within-class FA concentration is not significantly associated with CVD or T2D risk in the EPIC-Potsdam study (*P* value ≥ 0.05). CE, cholesteryl esters; CER, ceramide; DAG, diacylglycerol; DCER, dihydroceramide; HCER, hexosylceramide; LCER, lactosylceramide; LPC, lysophosphatidylcholine; LPE, lysophosphatidylethanolamine; MAG, monoacylglycerol; PC, phosphatidylcholine; PE, phosphatidylethanolamine; PEO, phosphatidylethanolamine ether; PEP, phosphatidylethanolamine plasmalogen; PI, phosphatidylinositol; SM, sphingomyelin; TG, triglycerides.

The associations between within-class FAs affected by the replacement of dietary SFA with UFA in the DIVAS RCT and the long-term CMD risk in the EPIC-Potsdam cohort study are presented in Figure 6. Overall, results from Cox proportional hazards models suggest that the MUFA-rich diet from the DIVAS study significantly decreased the concentrations of some FAs in DAG (i.e., 14:0, 15:0, 16:0, 18:0, and 22:4), TG (i.e., 14:1, 16:0, 17:0, 18:0, and 20:0), SM (i.e., 14:0 and 18:0), and HCER (i.e., 18:1) and that these lipid metabolites were all associated with higher CVD risk in the EPIC-Potsdam study. Reciprocally, the DIVAS MUFA-rich diet increased the concentrations of a within-class FA associated with lower CVD risk in the EPIC-Potsdam study (LCER [24:1]; HR = 0.73; 95% CI: 0.56, 0.94; *P* value = 0.02). However, the DIVAS MUFA-rich diet increased plasma levels of SM (24:1), a within-class FA associated with a greater CVD risk in the EPIC-Potsdam study (HR = 1.60; 95% CI: 1.27, 2.02; *P* value < 10^−4^). This beneficial synergy between the DIVAS MUFA-rich diet and long-term CVD risk in the EPIC-Potsdam study was consistent with the effects of the DIVAS MUFA/PUFA-rich diet, although fewer within-class FAs were affected by the latter dietary intervention arm. Finally, the lipidome-mediated links between the DIVAS dietary intervention and incident T2D risk in the EPIC-Potsdam study were not as clear, but they revealed that the DIVAS MUFA-rich diet decreased the concentrations of some of the within-class FAs that were strongly associated with T2D risk, such as TG (16:0; HR = 9.80; 95% CI: 3.96, 24.27; *P* value < 10^−6^), DAG (16:0; HR = 2.84; 95% CI: 1.75, 4.61; *P* value < 10^−4^), and DAG (18:0; HR = 2.22; 95% CI: 1.41, 3.51; *P* value < 10^−3^).

## Discussion

In this secondary analysis, lipidome-wide screening revealed broad reductions in SFA along with increased MUFA and PUFA concentrations across several plasma lipid classes (i.e., sphingolipids and glycerolipids) following the exchange of dietary SFAs with UFAs for 16 wk. These results might be partly explained by the absorption of dietary UFAs throughout the MUFA-rich and PUFA-rich dietary interventions, since glycerolipids are common FA storage structures found within lipoprotein particles such as chylomicrons [28,29]. In addition, this study is the first, to our knowledge, to report direct associations between dietary MUFA intakes and plasma PEP (18:1) levels. Plasmalogens (∼18% of all phospholipids) play an important role in modulating membrane fluidity and cell signaling and have scavenger properties, which protect the cell under conditions of oxidative stress [30,31]. C18:1 represents one of the major fatty alcohol species at the sn-1 position of plasmalogens and is proposed to be derived from dietary intakes or synthesized de novo [30]. In a case-cohort study from the PREDIMED RCT (*n* = 787), Wang et al. did not report any effect of a 1-y diet enriched in extra virgin olive oil on the plasmalogen lipid cluster score, which reflected the plasma concentrations of PEP and plasmalogen PC [32]. However, this lack of response in plasmalogen lipids might be partly explained by the high habitual consumption of olive oil within the Spanish cohort, which may have reduced the impact of the PREDIMED dietary intervention in a Spanish population relative to the UK DIVAS cohort. This highlights the importance of the background FA intakes in response to dietary fat interventions [33,34].

Other consequences of the DIVAS MUFA-rich diet included lower long-chain FA concentrations in plasma sphingolipids such as 18:1, 20:1, and 22:1 in CER and HCER after intervention compared to the SFA-rich diet. Although decreased MUFA concentrations in these lipid classes following the replacement of dietary SFAs with MUFAs might seem counterintuitive, these results are in line with experimental findings, which suggested that SFAs may be used as precursors for the first step of the endogenous synthesis of 16:1 and 18:1 sphingolipids catalyzed by the serine palmitoyltransferase enzyme [35]. Therefore, diets enriched in UFAs instead of SFAs might downregulate serine palmitoyltransferase activity, leading to lower endogenous synthesis of long-chain sphingolipids [36,37]. Evidence from human studies is sparse, but one cross-sectional study of 2860 Chinese participants observed similar results and reported inverse associations between dietary total PUFA intakes and plasma long-chain CER and HCER levels, although different lipid metabolite nomenclatures prevent the direct comparison of their results with those from the DIVAS study [38]. Similarly, 2 overfeeding RCTs reported decreased serum long-chain FAs (16:0, 18:0, 24:0, and 24:1) in CER and LCER following a PUFA-rich diet [39] and increased plasma long-chain CER levels following a SFA-rich diet [40].

Coordinated CLP analyses between DIVAS and EPIC-Potsdam revealed that both DIVAS UFA-rich diets reduced plasma concentrations of 15:0, 17:0, and 18:0 in glycerolipids (i.e., DAG and TG), which were associated with higher CVD risk in EPIC-Potsdam. Previous epidemiological studies on circulating odd-chain SFAs, used as biomarkers of dairy consumption [41], suggested their null or inverse associations with CVD risk [42], in contrast to the findings from EPIC-Potsdam [13]. However, most studies have measured odd-chain SFA within plasma/serum phospholipids or CE, or in total plasma/serum, whereas there is no previous epidemiological evidence of potential associations between specific DAG or MAG FA compositions and the CVD risk. Moreover, analyses from EPIC-Potsdam suggested direct associations between the CVD risk and some plasma sphingolipids (SM and HCER) containing 14:0, 18:0, and/or 18:1, which were sensitive to the DIVAS MUFA-rich diet. In support, a prospective cohort study of Chinese participants (*n* = 152/2627 CVD cases) found higher plasma HCER (18:1/16:0), HCER (18:1/18:0), SM (18:1/18:0), and SM (18:2/18:0) levels to be associated with higher risks of major CVD events [43]. Although the exact mechanisms underlying these associations have not been clarified, a cross-sectional analysis of 200 atherosclerotic carotid plaques reported higher SM concentrations from symptomatic patients [44], and further studies have that suggested dysfunctional SM synthesis and transport may be associated with atherosclerosis, valvular disease, and cardiomyopathy risks [45].

Overall, the DIVAS dietary interventions seemed less efficient in increasing plasma within-class FAs that are inversely associated with T2D risk. However, the MUFA-rich dietary intervention seemed to decrease the plasma levels of 16:0 in DAG and TG, along with 18:0 in DAG and SM, which were directly associated with T2D risk in EPIC-Potsdam [13]. Serum and plasma glycerolipids containing SFAs, especially 16:0 and 18:0, have been previously identified as potential predictors of the T2D risk in RCTs and prospective cohort studies [12,46,47], possibly by impairing insulin sensitivity pathways as suggested by animal and in vitro studies [48]. Furthermore, although recent findings from the PREDIMED RCT suggested inverse associations between plasma SM cluster scores and the T2D risk [12], other studies reported SFA-containing SM species may be associated with higher T2D risk and impaired insulin sensitivity [[49], [50], [51]]. In particular, a prospective cohort study of 2302 Chinese participants observed direct associations between the T2D risk and higher plasma levels of two 18:0-containing SM species: SM (16:1/18:0; HR = 1.45; 95% CI: 1.18–1.78) and SM (18:1/18:0; HR = 1.40; 95% CI: 1.17–1.68) [49].

In the DIVAS study participants, we identified that higher plasma levels of CE (18:0) and TG (12:0) were associated with higher LDL cholesterol, which was independent of the intervention group and other potential confounding factors. These results support previously described general lipid compositions of LDL particles, which contain ∼42% of CE and 6% of TG (wt/wt) [52]. In an overfeeding RCT (*n* = 36), a 3-wk SFA-rich diet led to higher concentrations of SFA-containing TG in LDL particles, which was associated with higher LDL susceptibility to oxidation and aggregation in the intima [53]. Furthermore, previous experimental and animal studies showed that dietary SFAs decrease hepatic LDL receptor expression, leading to increased circulating LDL cholesterol levels [54]. In addition, we identified that improved LDI Ach and LDI SNP, which are 2 estimates of microvascular reactivity, were associated with higher plasma PE (20:3) and CER (18:0) levels, respectively. The latter association contrasts with the findings from a recent case-control study of 90 patients with abnormal coronary endothelial function who displayed higher plasma CER (18:0) levels than controls [55], and more generally contrasts with studies suggesting the potential detrimental role of ceramides in atherosclerosis and CVD etiology [56,57]. Additionally, we observed direct associations between the arterial stiffness index measured by digital volume pulse and plasma LPC (i.e., 15:0 and 20:2) and those between night SBP and LPC (22:5) levels. Although previous studies reported direct associations between overall LPC and atherosclerosis or arterial stiffness [58,59], other findings suggest inverse associations between CVD risk markers and specific LPC molecular species [[60], [61], [62], [63]]. Finally, this secondary analysis revealed potential deleterious associations between MAG (12:0) and 24-h PP and those between CE (12:0) and P-selectin levels, but inverse associations between PC (14:1) and the day heart rate. Besides, 2 within-class FAs (i.e., TG [12:0] and CE [12:0]) significantly affected by the DIVAS dietary intervention were also associated with changes in CMD risk markers in fully adjusted linear regression models, which provides promising insights into the beneficial impact of replacing dietary SFAs with UFAs for CVD prevention. Overall, further studies are warranted to assess the strength of these associations and to decipher the potential roles of lipid classes and/or specific FAs in these relationships.

The strengths of this study include its extensive panel of investigated lipids, which were aggregated into within-class FAs to facilitate the interpretability of the findings. Second, this coordinated analysis between DIVAS and EPIC-Potsdam provided novel insights, bridging the gap between data from dietary intervention and prospective cohort studies. Finally, this analysis benefited from strong methodological approaches in the identification of lipidomic predictors of CMD risk and associated biomarkers by adjusting Cox proportional hazard models and linear regression models with a wide range of potential confounders, along with using crossvalidation methods and corrections from multiple testing. Nonetheless, some limitations of this study need to be acknowledged. First, although the CLP provided details on the acyl chains within lipid classes, there was no information on the location of the configuration of double bonds in FAs (i.e., cis or trans). Second, plasma-free FA levels were not available. Third, risk of lipid degradation and oxidation in plasma samples cannot be entirely ruled out, although the DIVAS samples had been stored at −80 °C and were not thawed until analysis [64]. There was no evidence that freeze-thaw cycles in the EPIC-Potsdam study samples had any impact on the observed associations with CMD risk [13]. Fourth, although previous studies have investigated the relationship between lipidomic patterns and health outcomes [32,65,66], this study focused on individual within-class FAs to better understand their role in CMD etiology. Finally, different approaches to measure and identify lipidomic species may impede the direct comparison of our findings with those from previous studies.

In conclusion, the replacement of dietary SFAs with UFAs in the DIVAS RCT led to modifications of the plasma lipidome metabolites among UK adults at the moderate CVD risk. Furthermore, our findings suggest that the associations between lipid metabolites and markers of CMD risk may be specific to certain FAs and lipid classes. Specifically, some sphingolipids and phospholipids may be related to novel CVD risk markers, including endothelial function, arterial stiffness, and ambulatory blood pressure. Finally, the coordinated lipidome-wide plasma screening performed in EPIC-Potsdam suggested that the changes in plasma lipid metabolites observed after the replacement of dietary SFAs with UFAs may be beneficially associated with long-term CMD risk. Overall, these results concur with current evidence on the benefits of replacing dietary SFAs with UFAs for CMD risk prevention and contribute to the evidence base on the role of lipid metabolites in CMD etiology.

## Data Availability

Data described in the manuscript and analytic code will be made available upon reasonable request pending application and approval from the corresponding author Professor Julie A. Lovegrove (j.a.lovegrove@reading.ac.uk).

## Appendix A Supplementary data

Multimedia component1 Multimedia component1
