# Supplementary material for: Replacement of dietary saturated with unsaturated fatty acids is associated with beneficial effects on lipidome metabolites: a secondary analysis of a randomized trial
Source: Am J Clin Nutr. 2023 Apr 11;117(6):1248–61. doi: 10.1016/j.ajcnut.2023.03.024 (PMC10315407; doi:10.1016/j.ajcnut.2023.03.024)
Supplement: Multimedia component1 [file mmc1.docx]

**Supplementary Table 1.** List of 28 fatty acids identified in lipidome-wide screening among participants from the DIVAS study (n=113).

| **Fatty acid shorthand notation** | **Fatty acid common name ^a^** |
| --- | --- |
| 12:0 | Lauric acid |
| 14:0 | Myristic acid |
| 14:1 | Myristoleic acid |
| 15:0 | Pentadecanoic acid |
| 16:0 | Palmitic acid |
| 16:1 | Palmitoleic acid |
| 17:0 | Hexadecenoic acid |
| 18:0 | Stearic acid |
| 18:1 | Oleic acid |
| 18:2 | Linoleic acid |
| 18:3 | α-linolenic acid |
| 18:4 | [Stearidonic acid](https://en.wikipedia.org/wiki/Stearidonic_acid) |
| 20:0 | [Arachidic acid](https://en.wikipedia.org/wiki/Arachidic_acid) |
| 20:1 | [Paullinic acid](https://en.wikipedia.org/wiki/Paullinic_acid) |
| 20:2 | Dihomo-linoleic acid |
| 20:3 | [Dihomo-γ-linolenic acid](https://en.wikipedia.org/wiki/Dihomo-%CE%B3-linolenic_acid) |
| 20:4 | Eicosatetraenoic acid |
| 20:5 | [Eicosapentaenoic acid](https://en.wikipedia.org/wiki/Eicosapentaenoic_acid) |
| 22:0 | [Behenic acid](https://en.wikipedia.org/wiki/Behenic_acid) |
| 22:1 | [Erucic acid](https://en.wikipedia.org/wiki/Erucic_acid) |
| 22:2 | Docosadienoic acid |
| 22:4 | [Docosatetraenoic acid](https://en.wikipedia.org/wiki/Docosatetraenoic_acid) |
| 22:5 | Docosapentaenoic acid (osbond acid) |
| 22:6 | [Cervonic acid](https://en.wikipedia.org/wiki/Cervonic_acid) |
| 24:0 | [Lignoceric acid](https://en.wikipedia.org/wiki/Lignoceric_acid) |
| 24:1 | [Nervonic acid](https://en.wikipedia.org/wiki/Nervonic_acid) |
| 26:0 | [Cerotic acid](https://en.wikipedia.org/wiki/Cerotic_acid) |
| 26:1 | Ximenic acid |

^a^ based on the most common double-bond *cis* configurations, as the complex lipid panel performed by Metabolon Inc. in this analysis did not allow for the resolution of double bond position or configuration.

**Supplementary Figure 1.** Flow-chart of participants from the DIVAS randomised controlled trial included the secondary analysis.

**
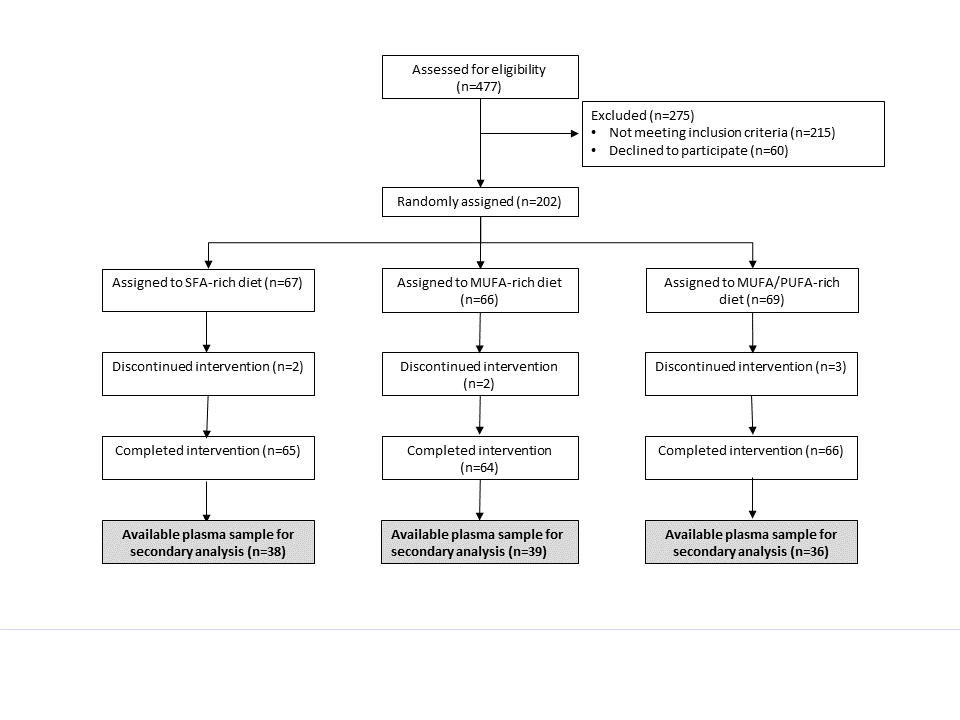
**

**Supplementary Figure 2.** Final regression coefficients (mean and SD) for the within-class FAs selected at least 9 times in the elastic-net regression models conducted among participants from the DIVAS study.

**Abbreviations:** CE cholesteryl esters; CER, ceramides; DAG, diacylglycerols; FA, fatty acid; HCER, hexosylceramides; LDI Ach, laser doppler imaging microvascular response to acetylcholine; LDI SNP, laser doppler imaging microvascular response to sodium nitroprusside; LDL-C, LDL cholesterol; LPC, lysophosphatidylcholine; LPE, lysophosphatidylethanolamine; MAG, monoacylglycerols; NEFAs, non-esterified fatty acids; PC, phosphatidylcholine; PE, phosphatidylethanolamine; PEP, phosphatidylethanolamine plasmalogen; PP, pulse pressure; QUICKI, quantitative insulin sensitivity check index; SBP, systolic blood pressure; TAG, triacylglycerols; TNF-α, tumour necrosis factor α.

**A**: Coefficients (mean and SD) for the within-class FAs selected at least 9 times in the elastic-net regression models on total cholesterol and LDL cholesterol (LDL-C) among participants from the DIVAS study.


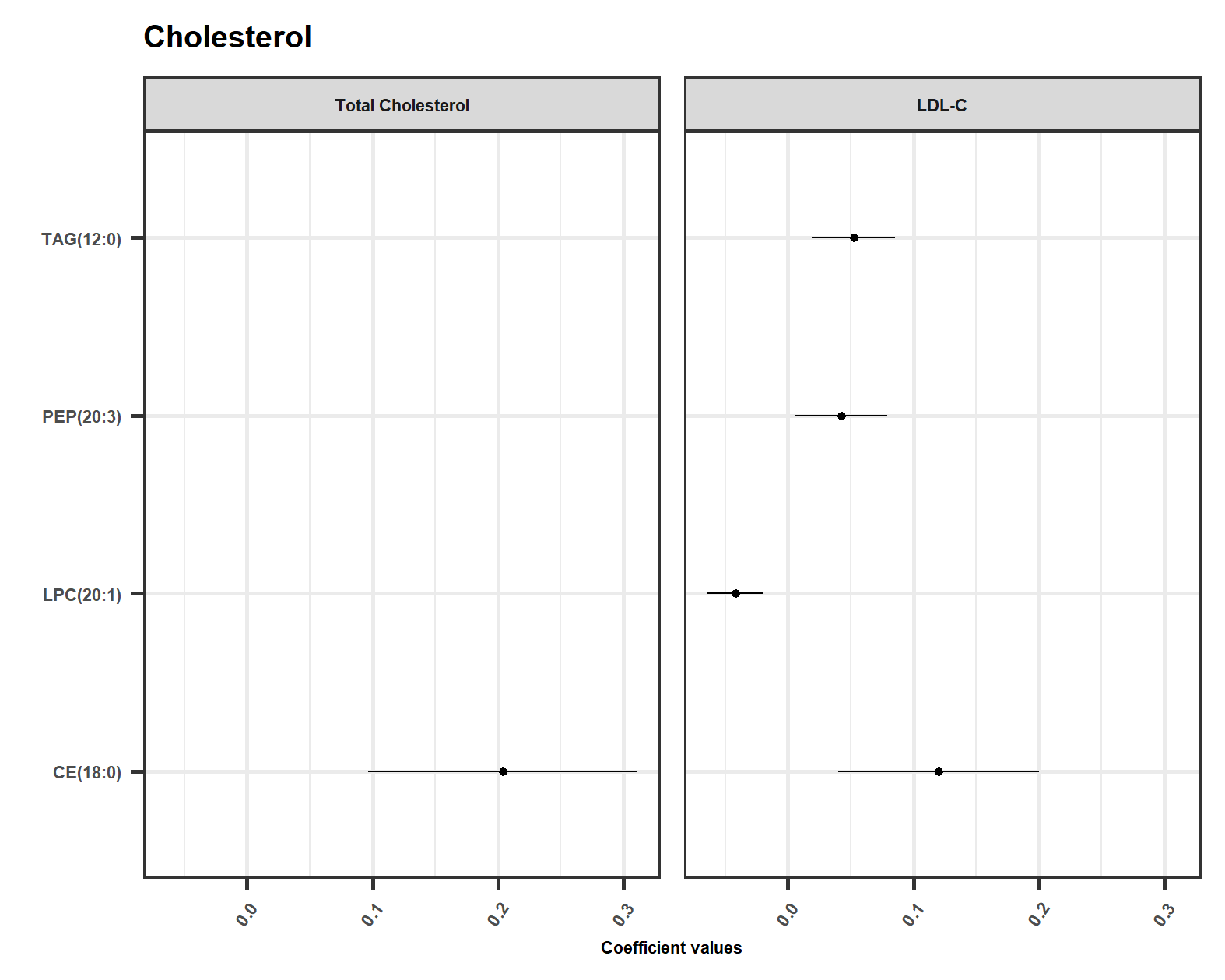


**A**

**B**: Coefficients (mean and SD) for the within-class FAs selected at least 9 times in the elastic-net regression models on non-esterified fatty acids (NEFAs) among participants from the DIVAS study.


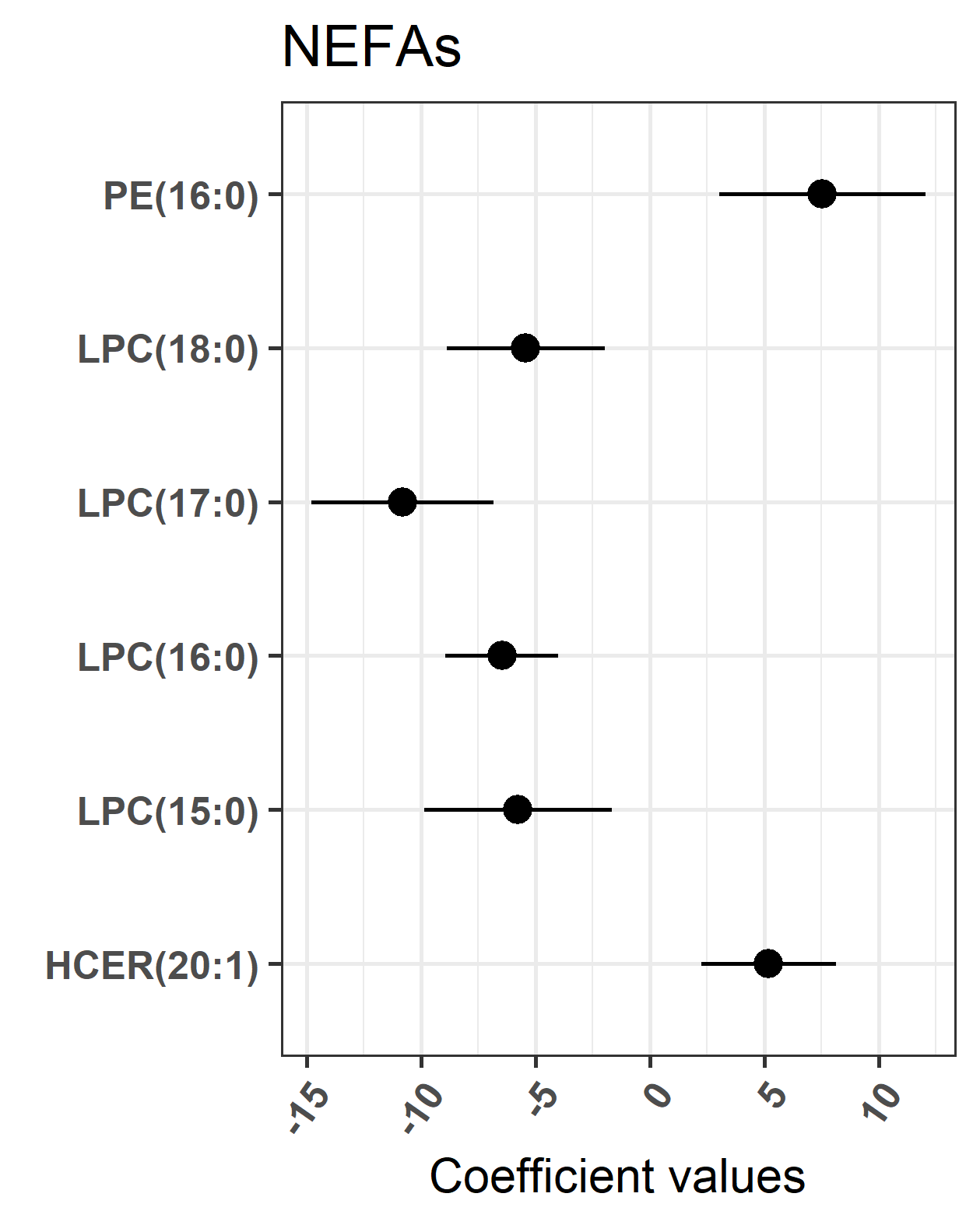


**B**

**C**: Coefficients (mean and SD) for the within-class FAs selected at least 9 times in the elastic-net regression models on quantitative insulin sensitivity check index (QUICKI) among participants from the DIVAS study.


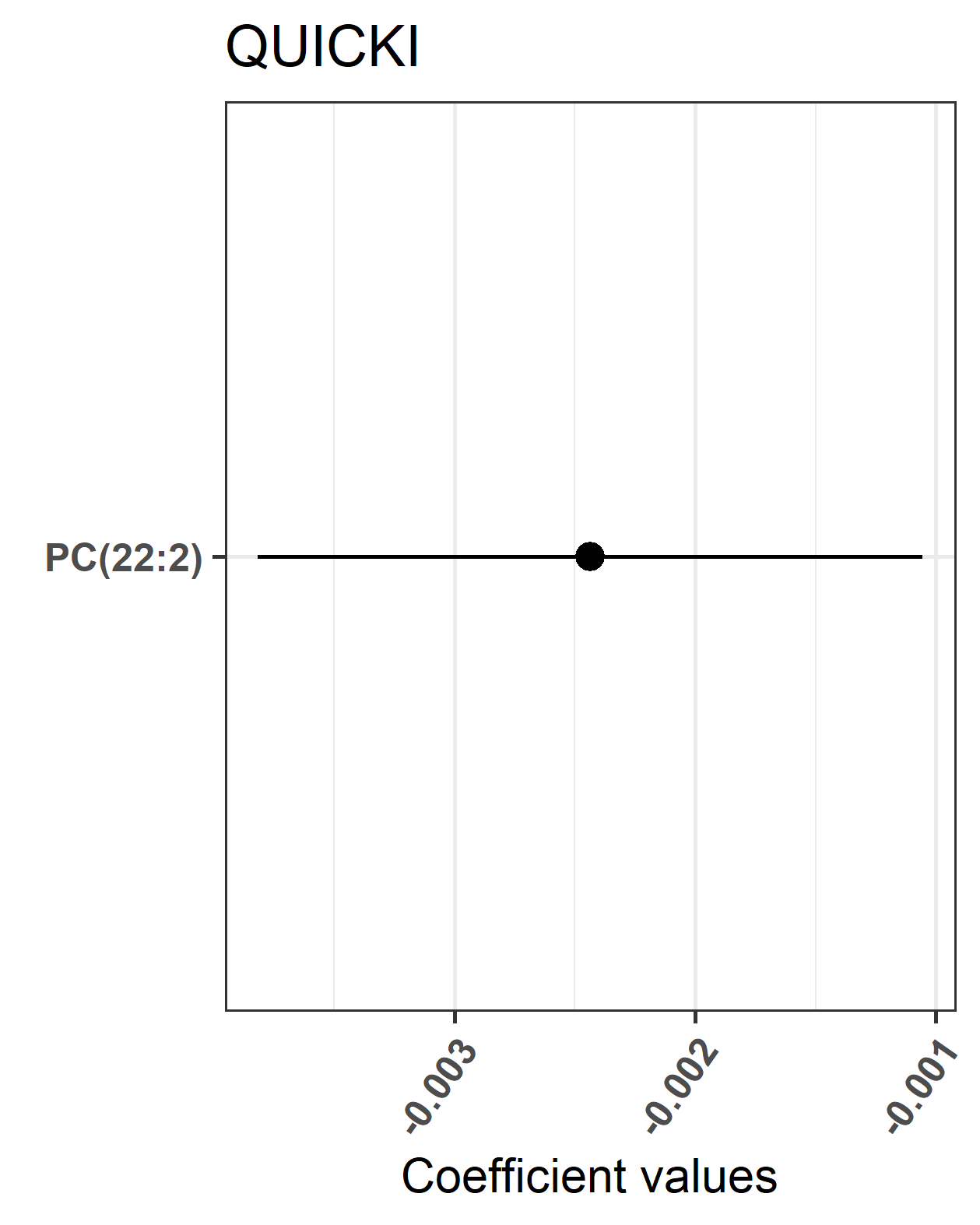


**C**

**D:** Coefficients (mean and SD) for the within-class FAs selected at least 9 times in the elastic-net regression models on laser doppler imaging microvascular response to acetylcholine (LDI Ach) and sodium nitroprusside (LDI SNP) among participants from the DIVAS study.


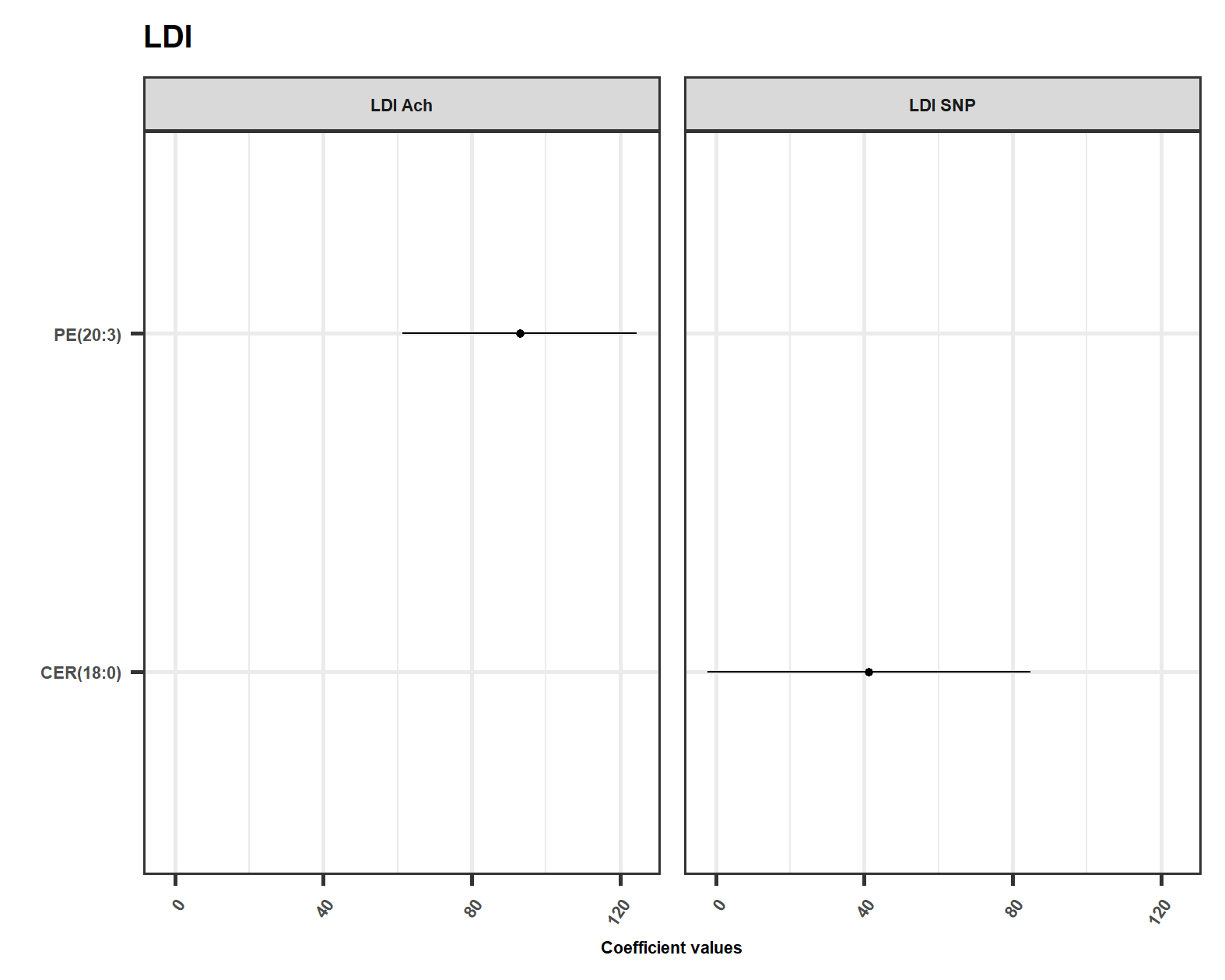


**D**

**E:** Coefficients (mean and SD) for the within-class FAs selected at least 9 times in the elastic-net regression models on reflection index, stiffness index, and pulse wave velocity among participants from the DIVAS study.


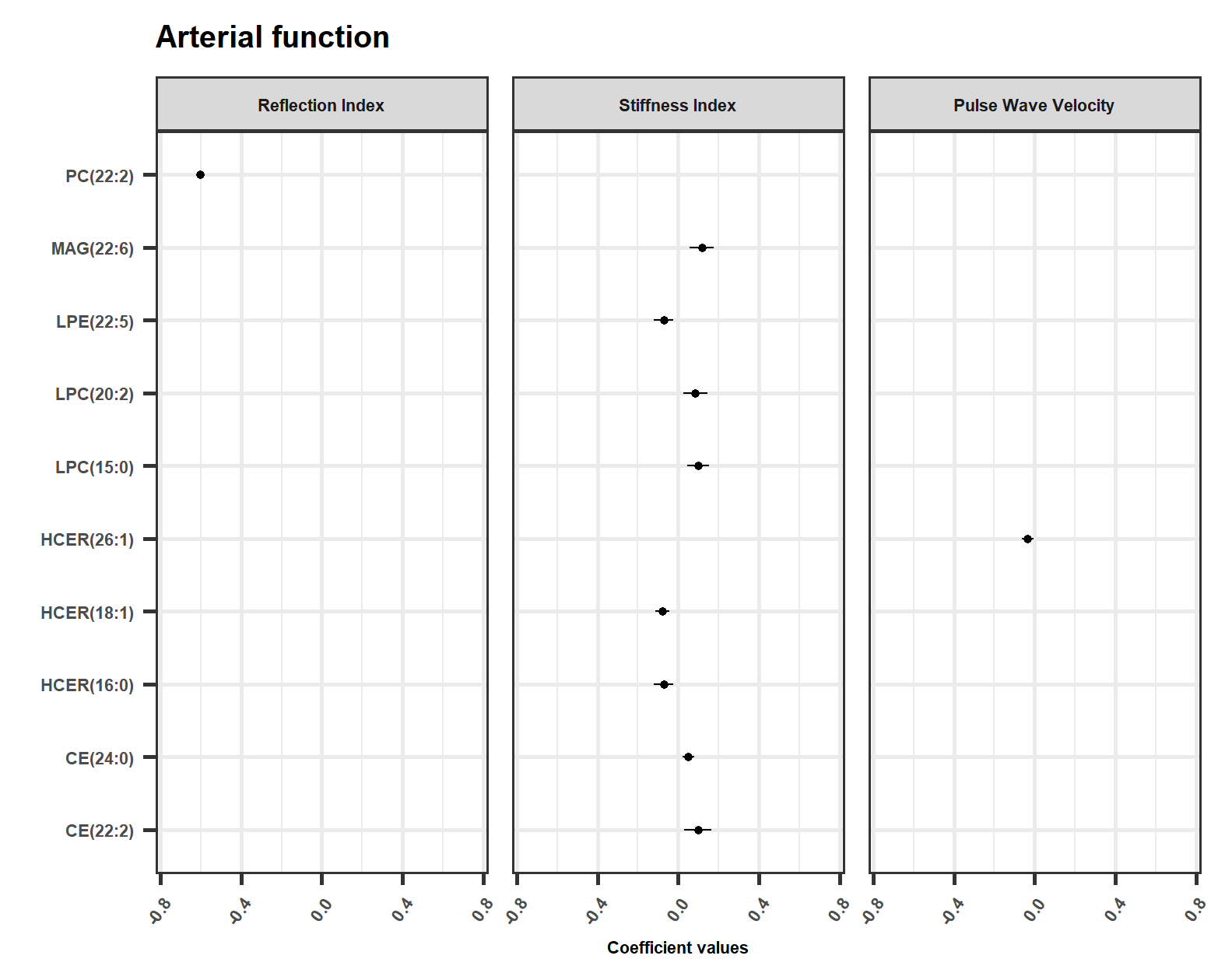


**E**

**F:** Coefficients (mean and SD) for the within-class FAs selected at least 9 times in the elastic-net regression models on night systolic blood pressure (SBP), 24h pulse pressure (PP), and day heart rate among participants from the DIVAS study.


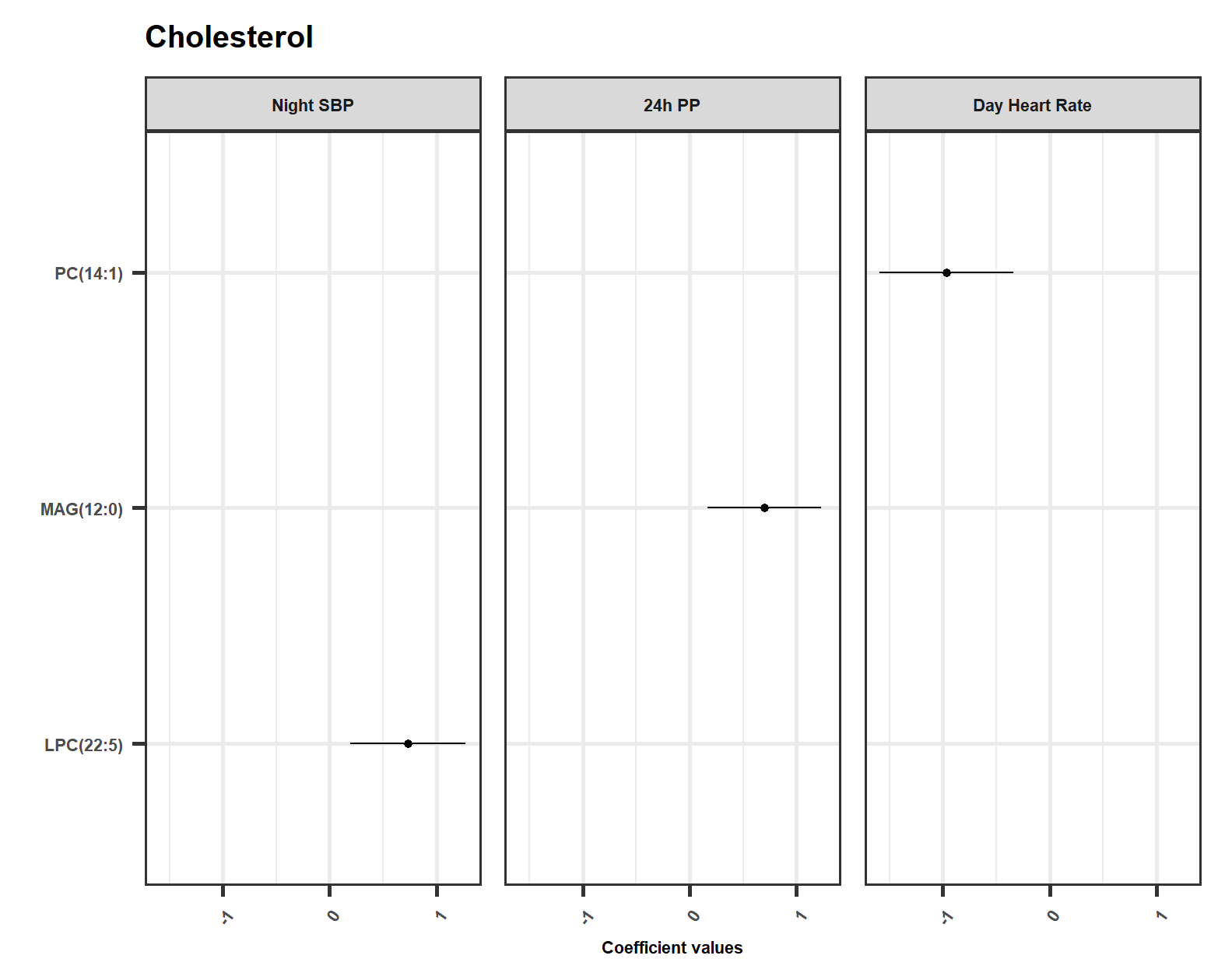


**F**

**G:** Coefficients (mean and SD) for the within-class FAs selected at least 9 times in the elastic-net regression models on tumour necrosis factor α (TNF-α) among participants from the DIVAS study.


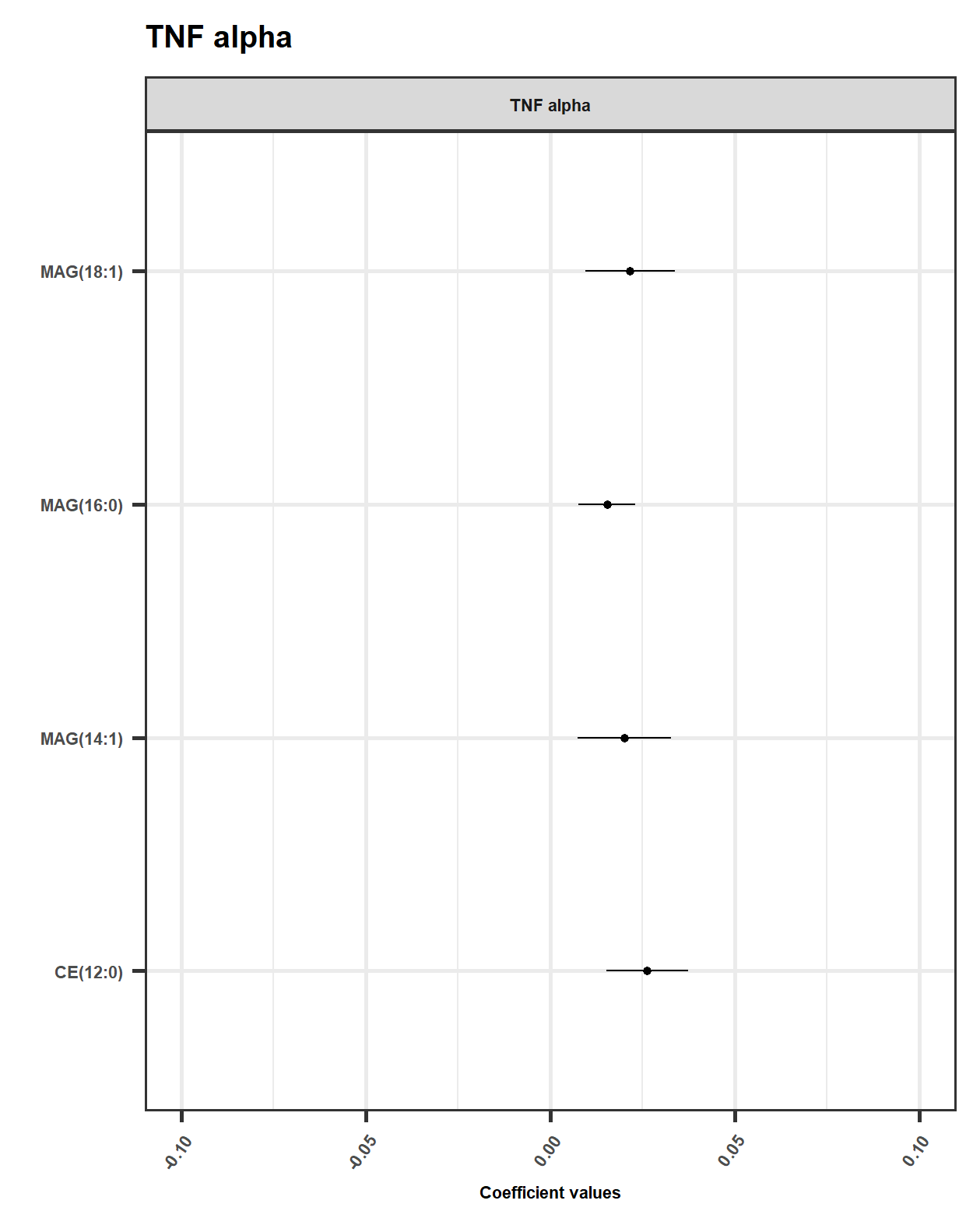


**GG**

**H:** Coefficients (mean and SD) for the within-class FAs selected at least 9 times in the elastic-net regression models on P-selectin among participants from the DIVAS study.


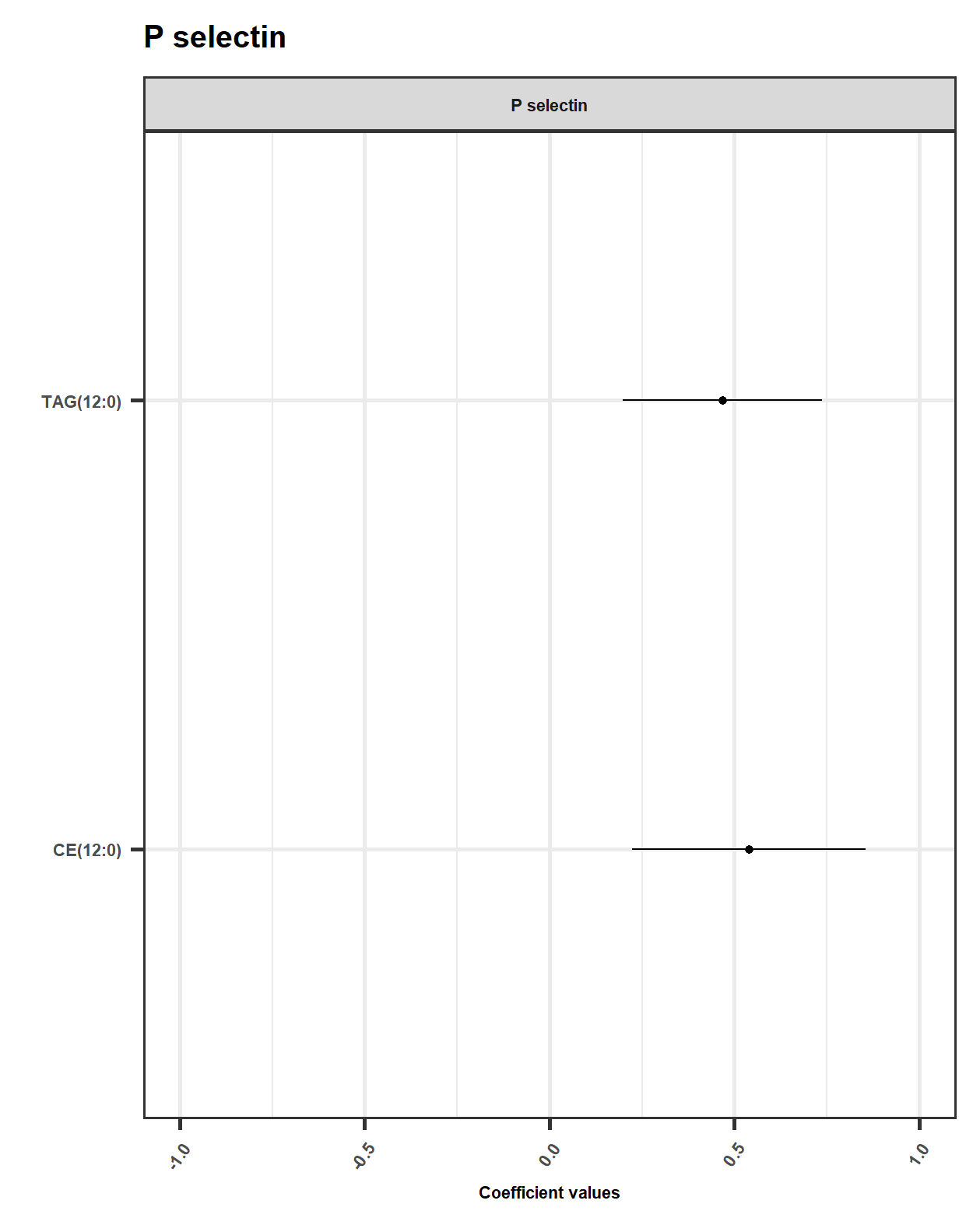


**HG**

**Supplementary Table 2.** Number of within-class FAs correlated with changes in CMD risk markers among participants from the DIVAS study, and Pearson correlation coefficients between multi-metabolite profiles and measured CMD risk markers.

| **CMD risk marker** | **Total within-class FAs consistently selected in ENR ^a^** | **Pearson correlation ^b^** | **95%CI** |
| --- | --- | --- | --- |
| **Total cholesterol** | 1 | 0.15 | 0.02 to 0.28 |
| **LDL cholesterol** | 4 | 0.21 | 0.08 to 0.33 |
| **NEFAs** | 6 | 0.26 | 0.14 to 0.38 |
| **QUICKI** | 1 | 0.23 | 0.09 to 0.36 |
| **LDI Ach** | 1 | 0.63 | 0.53 to 0.71 |
| **LDI SNP** | 1 | 0.60 | 0.50 to 0.69 |
| **Reflection Index** | 1 | 0.06 | -0.07 to 0.19 |
| **Stiffness Index** | 8 | 0.13 | -0.02 to 0.27 |
| **Pulse Wave Velocity** | 1 | 0.36 | 0.24 to 0.47 |
| **Night SBP** | 1 | 0.28 | 0.14 to 0.41 |
| **24h PP** | 1 | 0.25 | 0.10 to 0.39 |
| **Day heart rate** | 1 | 0.33 | 0.18 to 0.45 |
| **TNF-α** | 4 | 0.18 | 0.05 to 0.31 |
| **P-selectin** | 2 | 0.24 | 0.11 to 0.36 |

^a^ Number of within-class FAs selected at least 9 times in the 10-fold cross validation procedure for the elastic net regression approach.

^b^ The Pearson correlation coefficients reflect the correlation between the predicted change in CMD risk marker derived from the multi-metabolite models identified by the elastic net regression approach and the actual change in CMD risk marker measured during the DIVAS study (change = post-intervention – pre-intervention value).

**Abbreviations:** CE cholesteryl esters; CER, ceramides; CMD, cardiometabolic disease; DAG, diacylglycerols; FA, fatty acid; HCER, hexosylceramides; LDI Ach, laser doppler imaging microvascular response to acetylcholine; LDI SNP, laser doppler imaging microvascular response to sodium nitroprusside; LPC, lysophosphatidylcholine; LPE, lysophosphatidylethanolamine; MAG, monoacylglycerols; NEFAs, non-esterified fatty acids; PC, phosphatidylcholine; PE, phosphatidylethanolamine; PEP, phosphatidylethanolamine plasmalogen; PP, pulse pressure; QUICKI, quantitative insulin sensitivity check index; SBP, systolic blood pressure; TAG, triacylglycerol; TNF-α, tumour necrosis factor α.
